# Partial restoration of counterregulatory circulating glucagon by dapagliflozin and low-dose glibenclamide in men with type 1 diabetes: in vitro studies and randomised clinical crossover trial

**DOI:** 10.1016/j.ebiom.2026.106298

**Published:** 2026-05-19

**Authors:** Rui Gao, Ioannis Spiliotis, Haiqiang Dou, Thomas G. Hill, Lakshmi Kothegala, Caroline Miranda, Nkemjika Abiakam, Ruth L. Coleman, Sarah White, Amanda Adler, Patrik Rorsman

**Affiliations:** a Oxford Centre for Diabetes, Endocrinology and Metabolism, Radcliffe Department of Medicine, University of Oxford, Churchill Hospital, Oxford, UK; b Metabolic Physiology Unit, Department of Neuroscience and Physiology, University of Gothenburg, Gothenburg, Sweden; c NIHR Oxford Biomedical Research Centre, University of Oxford, Churchill Hospital, Oxford, UK

**Keywords:** Type-1 diabetes, Hypoglycaemia, Glucagon, Glibenclamide, Dapagliflozin, Glucose infusion rate

## Abstract

**Background:**

Hypoglycaemia, a common complication of insulin-treated diabetes, especially type 1 diabetes (T1D), is caused by impaired counterregulatory increases in plasma glucagon, leading to decreased hepatic glucose production. Alpha-cells in the pancreatic islets, which have sulfonylurea-sensitive ATP-regulated potassium (K_ATP_) channels, secrete glucagon at low plasma glucose levels in response to increased electrical activity. Activation of K_ATP_ channels inhibits alpha-cell electrical activity and consequently reduces glucagon secretion. Excessive secretion of somatostatin from neighbouring delta-cells (which are sensitive to the SGLT2 inhibitor dapagliflozin) also causes inappropriate paracrine inhibition of glucagon secretion at low glucose levels. We hypothesise that increased K_ATP_ channel activity and/or intra-islet somatostatin may contribute to the glucagon secretion defects seen in T1D and that these issues can be addressed with sulfonylureas or SGLT2 inhibitors.

**Methods:**

We first tested this hypothesis in vitro by comparing glucagon secretion in response to increasing concentrations of the sulfonylurea tolbutamide—used to titrate K_ATP_ channel activity—in isolated human islets from donors with T1D and healthy donors without a history of diabetes. Next, we conducted a single-centre, randomised, triple crossover, phase 2a superiority trial in people with T1D to evaluate the effectiveness of oral glibenclamide (0.3, 0.6, and 3 mg/day, over 14–18 days per dose) or an acute single dose of dapagliflozin (10 mg) in restoring glucagon counter-regulation. This was assessed using a hyperinsulinaemic-hypoglycaemic clamp in 12 male participants with T1D, with responses from 6 healthy controls (4 males and 2 females) serving as a reference. The primary endpoint was the change in plasma glucagon levels during hypoglycaemia from baseline (no medication) to post-treatment (glibenclamide or dapagliflozin). All participants were included in the analyses. The study is registered with ISRCTN (58098350).

**Findings:**

In islets from 9 healthy male donors, lowering glucose from 6 to 1 mM (to emulate hypoglycaemia) stimulated glucagon secretion by 120%. This effect was not observed in islets from 7 donors with T1D (4 males and 3 females). In these islets, a low concentration (10 μM) but not a high (100 μM) concentration of the sulfonylurea tolbutamide stimulated glucagon release by 70%. During the trial, insulin-induced hypoglycaemia (plasma glucose: <3 mM), in the absence of glibenclamide or dapagliflozin, produced a >700% increase in circulating glucagon in the control group, but only a 54% increase in participants with T1D. The latter value fell to <10% in the 8 participants with low circulating C-peptide levels (≤6 pM). In these participants, glibenclamide (0.3–0.6 mg/day for 14–18 days) raised circulating glucagon during hypoglycaemia by 125–150%; this correlated with reduced glucose infusion requirements, reflecting stimulation of endogenous glucose production. No effect on either parameter was seen with 3 mg/day glibenclamide. These increases in circulating glucagon were associated with better glucose control, as measured by continuous glucose monitoring during the last 7 days of each treatment period. Similar effects on plasma glucagon secretion and glucose infusion rates were observed with the SGLT2 inhibitor dapagliflozin.

**Interpretation:**

Enhanced K_ATP_ channel activity in alpha-cells and/or increased paracrine inhibition by somatostatin impairs the counterregulatory glucagon response in T1D. These issues can be partially corrected by glibenclamide or dapagliflozin. We suggest that these agents be further tested as adjuvants to insulin therapy in T1D to help reduce the occurrence of iatrogenic hypoglycaemia and the risks it poses.

**Funding:**

Supported by The Leona M. and Harry B. Helmsley Charitable Trust. The funding body did not influence the study's design, data analysis, or interpretation.

**Research in context**

**Evidence before this study**

In type-1 diabetes (T1D), glucagon secretion becomes impaired, increasing the risk of severe, potentially fatal hypoglycaemia associated with insulin therapy. The mechanisms behind this are unknown; in vitro experiments suggest that metabolic disturbances in alpha cells that produce glucagon increase the activity of ATP-sensitive potassium (K_ATP_) channels, which, in turn, reduce glucagon secretion. These channels are inhibited by sulfonylureas (such as glibenclamide), drugs commonly used in clinical practice to improve insulin secretion in type-2 diabetes (T2D). The SGLT2 inhibitor dapagliflozin raises circulating glucagon levels in people with T2D. In addition, residual beta-cell function (monitored as circulating C-peptide) lowers the risk of hypoglycaemia, an effect that has been attributed to loss of a suppressor function on delta-cells by beta-cells via electrical coupling.

**Added value of this study**

We demonstrate that glibenclamide, a sulfonylurea, at doses less than 10% of those used in T2D to increase insulin, can be repurposed to improve counterregulatory glucagon in people with T1D. This approach reduces the glucose infusion requirement (indicating stimulation of endogenous glucose production) and enhances glycaemic control. Similar results were seen with dapagliflozin.

**Implications of all the available evidence**

Dapagliflozin and glibenclamide, by increasing circulating glucagon levels, may provide therapeutic benefits as adjuncts to insulin therapy in T1D when tested with clinically relevant endpoints.

## Introduction

Plasma glucose is controlled by a dynamic balance between circulating insulin (produced by the beta-cells of the pancreatic islets), which lowers plasma glucose, and glucagon (secreted by the islet alpha-cells), which raises plasma glucose.^1^ Normally, when plasma glucose drops below 5 mM (90 mg/dl), it triggers an increase in circulating glucagon.^2^ This response helps restore normal blood glucose levels by stimulating hepatic glucose production. For reasons that remain unknown, this life-saving mechanism becomes defective in many people with type-1 diabetes (T1D)^3^; this increases the risk of hypoglycaemia linked with insulin treatment.^4^ People with T1D experience, on average, two episodes of symptomatic hypoglycaemia every week.^5^ Because of hypoglycaemia risk, insulin treatment in some people with T1D may be less aggressive than necessary for optimal glycaemic control. Despite this, nearly 10% of all patients with T1D eventually succumb to hypoglycaemia.^6^

Glucagon secretion is normally controlled by complex crosstalk between intrinsic (within the alpha-cell itself) and paracrine processes (mediated by factors like somatostatin, released by neighbouring delta-cells)^7^ as well as electrical signals between the beta- and the delta-cells.^8^ Glucagon secretion is also controlled by neuronal factors, such as acetylcholine and adrenaline released by nerve endings within the islets, as well as circulating hormonal factors like the gut hormones gastric inhibitory polypeptide (GIP) and glucagon-like peptide-1 (GLP-1).^9^, ^10^, ^11^ Alpha-cells have ATP-regulated potassium (K_ATP_) channels that, at the molecular level, are identical to those in beta-cells, where their glucose-induced closure initiates action potential firing and insulin secretion.^12^ According to one hypothesis, an elevation in blood glucose lowers glucagon secretion by inhibiting K_ATP_ channels.^7^ Because alpha- and beta-cells have different sets of voltage-gated ion channels, closing K_ATP_ channels and the resulting decrease in membrane potential from −55 to −45 mV (called ‘membrane depolarization’) lowers electrical activity in alpha-cells but increases it in beta-cells. This difference explains why glucose inversely regulates the secretion of these two hormones: in people without diabetes, pharmacological inhibition of K_ATP_ channels with tolbutamide lowers plasma glucagon but raises plasma insulin.^13^ Paradoxically, diazoxide, which activates the K_ATP_ channel, also lowers circulating glucagon under hypoglycaemic conditions.^14^ Collectively, these data suggest that: i) there is a bell-shaped relationship between K_ATP_ channel activity and glucagon secretion; ii) glucagon secretion only occurs within a narrow range of K_ATP_ channel activity; and iii) activity outside this range (either higher or lower) inhibits glucagon secretion.^7^

K_ATP_ channel blockers (like tolbutamide and glibenclamide) have previously been reported to stimulate glucagon secretion under normoglycaemic conditions in people with T1D, but their effects at low glucose were not studied.^15^^,^^16^ We now hypothesise that increased K_ATP_ channel activity impairs counterregulatory glucagon secretion in T1D. We tested this by a combination of in vitro studies on isolated islets (from cadaveric donors with or without T1D) and a clinical trial. The trial was designed to determine whether treating people with T1D with different dosages of the oral sulfonylurea glibenclamide improves the counterregulatory glucagon response during hyperinsulinaemic hypoglycaemic clamps. We also compared the effects of glibenclamide with dapagliflozin, which has previously been reported to increase plasma glucagon.^17^^,^^18^ Counterregulatory plasma glucagon levels in participants with T1D under baseline conditions and following treatment with glibenclamide or dapagliflozin are compared with those seen in healthy controls (without medication).

## Methods

### Pancreatic islet donors

We obtained human pancreatic islets, with appropriate ethical approval, from the Nordic Network for Clinical Islet Transplantation^19^ or the Alberta Diabetes Institute (ADI) Isletcore.^20^ Supplementary Table S1 summarises the characteristics of the human islet donors. The isolated islets were cultured in sterile suspension dishes in RPMI1640 (Cat# 11879020, Gibco, Waltham, Massachusetts, US) medium containing 5.5 mM glucose, 10% foetal bovine serum (FBS, Cat# F7524, Sigma–Aldrich, St. Louis, Missouri, US), and 1% penicillin/streptomycin (Cat# 15140122, Gibco) at 37 °C in a 5% CO_2_ incubator for 24–72 h before the hormone secretion experiments.

### In vitro glucagon secretion

Human islets were preincubated at 37 °C in Krebs–Ringer Bicarbonate buffer (KRBB) containing 0.1% Bovine Serum Albumin (BSA, Cat# A6003, Sigma–Aldrich) and (in mM) 140 NaCl, 4.7 KCl, 2.5 CaCl_2_, 1.1 KH_2_PO_4_, 1.2 MgSO_4_, 25 NaHCO_3_, 10 HEPES (pH 7.4 with NaOH), and 6 d-glucose for 1 h. Groups of 10 size-matched islets were then transferred into 200 μl KRBB (0.1% BSA) and incubated at 1 or 6 mM glucose with either 10 or 100 μM tolbutamide (Cat# T0891, Sigma–Aldrich), dissolved in DMSO to a final concentration of 0.01–0.1%. Tolbutamide and glibenclamide both block K_ATP_ channels, but glibenclamide is several orders of magnitude more potent and, unlike tolbutamide, shows significant intracellular uptake and poor reversibility.^21^^,^^22^ Therefore, tolbutamide is preferred for in vitro studies.

Glucagon was measured using a highly specific and sensitive ELISA (Cat# 10-1271-01, RRID:AB_2737304, Mercodia AB, Uppsala, Sweden). Insulin was measured using a human insulin ELISA (Cat# 10-1113-01, RRID:AB_2877672, Mercodia AB, Uppsala, Sweden). Somatostatin was measured by radioimmunoassay (Cat# RB306RUO, DIAsource ImmunoAssays, Louvain-la-Neuve, Belgium).

### Clinical trial design

#### Study design

LEGEND-D (Low dosE GlibENclamide and Dapagliflozin in type 1 Diabetes) is an exploratory (‘proof-of-concept’), randomised, open-label, triple crossover phase 2a trial (ISRCTN58098350, https://www.isrctn.com/ISRCTN58098350?q=58098350&filters=&sort=&offset=1&totalResults=1&page=1&pageSize=10, date of registration: 25/09/2023) designed to determine whether treating people with T1D for 14–18 days with three different doses of glibenclamide results in differences, compared to baseline (when no participant took glibenclamide), in the increase of plasma glucagon during induced hypoglycaemia. Tolbutamide is only available as an oral tablet (typically 500 mg). As a first-generation sulfonylurea, it is less potent than second-generation agents (like glibenclamide). The only liquid formulation of glibenclamide that allows accurate dosing with a syringe (1–5 ml) is Amglidia (ATC code: A10BB01, EMA product number: EMEA/H/C/004379, manufactured by AMMTeK, Paris, France), available at a fixed concentration of 0.6 mg/ml. There is no equivalent liquid placebo with the same taste, nor is there an alternative formulation at a different concentration. The use of tablets was also avoided, as pill splitting is inaccurate and therefore unsuitable for achieving a 0.3 mg dose (equivalent to 1/16 of a commercially available 5 mg tablet). Therefore, a blinded design is technically difficult to implement in this study. To address this limitation, we adopted a crossover design to minimise confounding effects due to treatment sequence and dosage. Responses are compared to those observed in healthy participants. The primary endpoint was the plasma glucagon concentration at 40 min during the hypoglycaemic phase of the hyperinsulinaemic hypoglycaemic clamp. Study visits took place at the Clinical Research Unit (CRU) in the Oxford Centre for Diabetes, Endocrinology, and Metabolism. The trial was approved by the South West - Central Bristol Research Ethics Committee (reference 23/SW/0098). In the context of the pandemic, there was no patient or public involvement in the design of the trial. Nor were patients or the public involved in the conduct or reporting of the trial.

**Fig. 1:**
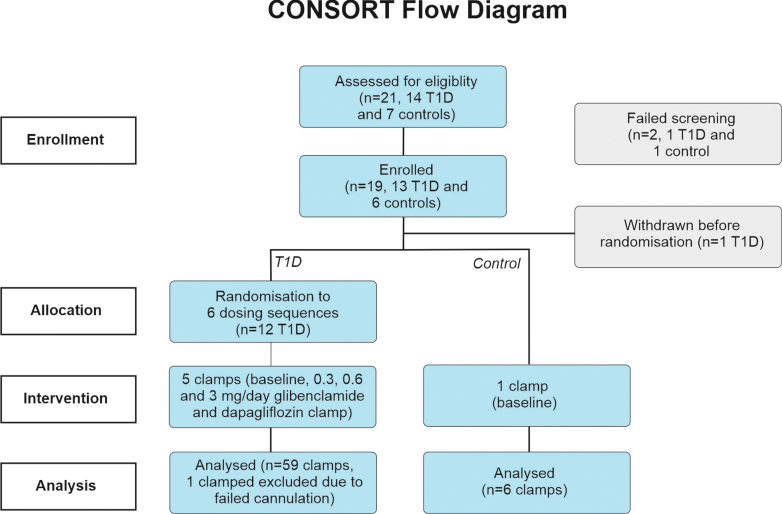
**Consort diagram of LEGEND-D trial**.

A CONSORT flow diagram illustrating the progression through the trial phases for the two groups is shown in Fig. 1. Following consent and screening, participants underwent a baseline clamp without investigational medication to measure plasma glucagon under basal, euglycemic, and hypoglycaemic conditions. Participants were then randomised to six sequences of glibenclamide dose steps using an independent computer-based randomisation schedule (Sealed Envelope Ltd, London, UK). Members of the study staff did not have access to the sequence prior to assignment. Each step lasted 14–18 days and was immediately followed by a clamp procedure (Supplementary Figure S1). Participants with T1D self-administered Amglidia at 0.3, 0.6, or 3 mg/day (dose split into twice daily, i.e., BID), with at least 2 days of washout period between each step. Compliance with treatment was checked at each CRU visit. Finally, a single 10 mg dose of dapagliflozin (Forxiga, ATC code: A10BK01, EMA product number: EMEA/H/C/002322, manufactured by AstraZeneca, Cambridge, UK) was given by CRU staff to participants with T1D approximately 2 h before the final clamp. At this dose, dapagliflozin increases to a peak concentration of 370 nM within 2 h.^23^ Our previous electrophysiological measurements indicate a rapid onset of dapagliflozin's effects,^18^ and it was not deemed necessary to test a range of doses because it does not exhibit the complex dose-dependent effects on glucagon secretion previously documented for sulfonylureas in vitro.^23^^,^^24^ Dapagliflozin use was not randomised, but baseline glucagon levels remained within ±13% throughout the study (Supplementary Figure S2). Participants could withdraw at any point during the trial. A control group without diabetes mellitus was also included to see how plasma glucagon responds to euglycaemia and hypoglycaemia in healthy individuals. These participants did not receive any investigational medication.

#### Participants

Participants included individuals with T1D and those without diabetes (‘controls’). They were recruited through the CRU from diabetes outpatient clinics at Oxford University Hospitals and Milton Keynes University Hospital, as well as via local advertisements. Eligibility criteria included a T1D duration of at least 12 months, treatment with either an insulin pump (continuous subcutaneous insulin infusion) or multiple daily insulin injections, an HbA1c below 10% (86 mmol/mol) at screening, and age between 18 and 75 years. Exclusion criteria included recent diabetic ketoacidosis within the past month; severe hypoglycaemia requiring third-party assistance within the past 12 months; active diabetic retinopathy; a history of seizures, ischaemic heart disease, heart failure, or stroke; known malignancy; current pregnancy or breastfeeding; current use of oral corticosteroids; or participation in another clinical trial within the past 3 months. Additionally, individuals with anaemia, impaired renal function, or abnormal liver function tests were excluded. Written informed consent was obtained from all participants.

Between March and November 2024, 21 participants were screened. After 4 screen failures and 1 withdrawal before the baseline clamp, 12 participants with T1D (aged 23–64 years; HbA1c 54.5 ± 2.2 mmol/mol) and 6 non-diabetic controls (aged 34–56 years; HbA1c 33.7 ± 1.2 mmol/mol) were enrolled and included in the final analyses. Age and BMI were similar between the two groups. The average duration of T1D was 28 years (range: 2–59 years; median: 24 years), and the age at onset was 20 years (range: 2–44 years; median: 20 years). Residual beta-cell function (serum C peptide: >30 pM) may provide some protection against hypoglycaemia^25^; in our study, C-peptide was undetectable (<3 pM) in 7 of the participants with T1D, 6 pM in an 8th T1D participant, and ≥30pM in 4 participants. In these latter four participants, the concentration averaged 81 ± 30 pM (range: 30–163 pM). No participants had C-peptide levels between 6 and 30 pM. Participant baseline characteristics are summarised in Table 1.

**Table 1:** Trial participant baseline characteristics.

Characteristic	Type 1 diabetes (N = 12)	Non-diabetic control (N = 6)
Age (years)	48.0 ± 3.6	45.8 ± 3.5
Sex	12 males, 0 females	4 males, 2 females
Body-mass index (kg/m^2^)	27.3 ± 1.3	28.1 ± 1.1
HbA1c (mmol/mol)	54.5 ± 2.2	33.7 ± 1.2
Duration of diabetes (years)	28.3 ± 5.2	–
Age at onset of diabetes (years)	20.3 ± 3.5	–
Insulin therapy		
Multiple daily injections (%)	10 (83.3%)	–
Insulin pump (%)	2 (16.7%)	–
Daily insulin dose (units)	56.2 ± 6.5	–
Plasma C-peptide		–
<30 pM (%)	8 (66.7%)	–
≥30 pM (%)	4 (33.3%)	–
Hypoglycemia awareness (Gold score)	2.25 ± 0.25 Range: 1–4^a^	–

a Score ≤2: Normal awareness of hypoglycaemia; Score 3: Borderline awareness; Score ≥4: Impaired awareness of hypoglycemia.

#### Hyperinsulinaemic-euglycaemic–hypoglycaemic clamps

The design of the clamp studies is summarised in Supplementary Figure S3. Each clamp started at 0800 h after an overnight fast from midnight; participants with T1D also omitted their morning fast-acting insulin bolus. Participants were asked to avoid any exercise and to consume their usual diet for 3 days before each study visit. We inserted two intravenous lines in each participant: 1) in an antecubital vein for infusion of 20% dextrose at a variable rate and human soluble insulin (Actrapid, ATC code: A10AB01, EMA product number: EMEA/H/C/000424, manufactured by Novo Nordisk, Bagsværd, Denmark) at a fixed rate (0.5 mU·kg^−1^min^−1^); and 2) in a distal vein of the contralateral hand or arm wrapped in a warm blanket for blood sampling. Intravenous dextrose was adjusted every 5 min based on blood glucose (measured using HemoCue Hb 201^+^, Ängelholm, Sweden) to maintain target blood glucose concentrations at approximately 6.0 mM (euglycaemic phase) for 40 min, followed by lowering to about 2.8 mM (hypoglycaemic phase) for another 40 min. Arterialised venous samples for measuring glucagon and somatostatin were collected at baseline (i.e., without any exogenous insulin) and at 0, 15, 30, and 40 min after euglycaemia or hypoglycaemia was achieved. At the end of the clamp, the insulin infusion was discontinued, and normoglycaemia was restored. Blood ketone levels were measured at the end of the clamps following dapagliflozin treatment using the FreeStyle Optium Neo Blood Glucose and Ketone Monitor (Abbott, Illinois, US).

#### Continuous glucose monitoring (CGM)

All participants with T1D were given and wore masked (blinded) CGM sensors (Freestyle Librae 2, Abbott) during the trial, with education provided by trained research staff. We used the one-week CGM data before each clamp (representing steady-state medication) for analysis of glycaemic control.

#### Sample handling and biochemical assays

Blood samples were collected in chilled EDTA-containing tubes, centrifuged at 4 °C, and stored at −80 °C. Plasma glucagon was measured using ELISA (Cat# 10-1271-01, RRID: AB_2737304, Mercodia AB) and somatostatin by radioimmunoassay (Cat# RB306RUO, DIAsource ImmunoAssays). Serum C-peptide was analysed with electrochemiluminescence immunoassay (Roche Diagnostics E170 analyser, Indianapolis, USA), and glucose was measured using the glucose oxidase method (Abbott Architect c8000). Both tests were performed locally by the Department of Clinical Biochemistry, Oxford University Hospitals.

#### Endpoints

The primary endpoint was the change in plasma glucagon levels during hypoglycaemia from baseline to post-glibenclamide or post-dapagliflozin treatment (both relative and absolute) in participants with T1D. We note that this should be considered a surrogate marker rather than a clinical outcome, although increased glucagon is expected to reduce the risk of hypoglycaemia. Predefined exploratory endpoints included plasma somatostatin levels (in pM), blood glucose levels (in mM), and serum C-peptide levels (in pM). Other exploratory endpoints involve comparing the change in plasma glucagon (in pM) in T1D to that of non-diabetic controls; changes in time in range (TIR) assessed by CGM in participants with T1D; and the effect of treatment on hepatic glucose production (measured as glucose infusion rate; in mg per min and per kg of body weight) in participants with T1D.

### Statistics

To calculate the sample size, since there are no data on plasma glucagon levels from human trials using low-dose sulfonylureas in patients with T1D, we based the calculations on data from the LEGEND-A trial (NCT02830048 - completed)^26^ and interim analysis from the DEPTH trial (NCT03537131—suspended due to the COVID-19 pandemic). The LEGEND-A trial indicated that glibenclamide (0.3 mg/day) might reduce plasma glucagon levels by about 30% (effect size) in participants with type 2 diabetes. Meanwhile, the DEPTH trial found that participants with T1D who experienced exercise-induced hypoglycaemia had mean plasma glucagon levels of 5.5 ± 2.1 pM (mean ± S.D.) at hypoglycaemia onset, defined as plasma glucose below 3.3 mM. In contrast, data from a clinical study involving healthy volunteers[5], using similar methods and a comparable plasma glucagon assay, showed mean plasma glucagon levels of 23.3 ± 5.7 pM during the hypoglycaemia plateau. These figures suggest that, with 20 participants with T1D and 10 without diabetes (accounting for 5% dropout), the LEGEND-D trial would have 80% power to detect a 30% increase in plasma glucagon levels—the primary endpoint—during induced hypoglycaemia induced by a hyperinsulinaemic-hypoglycaemic clamp.

We acknowledge that we did not reach the originally intended sample size. This was due to slower-than-expected recruitment caused by various factors, including the demanding nature of the protocol, which required multiple prolonged clamp procedures and washout periods. Additionally, this physiological proof-of-concept study was conducted within a fixed funding period and timeline. When it became clear that we could not achieve the planned sample size within these constraints, recruitment was halted. Notably, the funding was insufficient for a larger study because the original budget (pre-Covid) did not include the now-mandatory involvement of a registered trials unit, which doubled the study's cost. Clearly, the 40% reduction in sample size did not affect the ability to detect the primary endpoint (an increase in glucagon), indicating a sizeable effect. Although fewer participants will reduce power, we did not - consistent with good statistical practice^27^ - perform a post-study power calculation.

We did not initially plan a priori subgroup analyses; any post hoc subgroups should be considered as exploratory and hypothesis-generating. It has been reported that plasma C-peptide levels ≥30 pM confer some protection against hypoglycaemia.^25^ We therefore chose to compare the effects of glibenclamide and dapagliflozin on plasma glucagon in participants whose C-peptide levels were ≤6 or ≥30 pM. Comparing plasma glucagon levels during hypoglycaemia across these subgroups may provide additional mechanistic insights.

The primary null hypothesis was that there is no difference in counter-regulatory plasma glucagon concentration relative to baseline (pre-treatment) in people randomised to glibenclamide at each of the three dosages. We used generalised linear mixed effects models with fixed dose and random subject effects to estimate changes in circulating glucagon (pM) with different dosages of glibenclamide, with a further model including fixed effects for treatment period, treatment sequence, age, body mass index (BMI), blood pressure, and heart rate.^28^ The general univariate linear mixed model is described by the formula

Y=Xβ+Zb+ϵ

where ***Y*** is the *n* × 1 random response vector, ***X*** is the *n* × (*p* + 1) design matrix for the fixed effects, ***β =*** (*β*_0_, *β*_1_, … …, *β*_*p*_)^T^ is the (*p* + 1) × 1 coefficient vector of fixed effects, ***b*** is a *q* × 1 vector of random effects, ***Z*** is the associate matrix design for the random effects, and ***ϵ*** is the *n* × 1 vector of random errors. It assumes that ***ϵ*** is a random vector with a mean of **0** and a variance–covariance matrix ***R***, ***b*** is a random vector with a mean of **0** and a variance–covariance matrix ***D***, and a null variance–covariance matrix between ***ϵ*** and ***b***, Cov(***ϵ***, ***b***) = **0**_*n*__×__*q*_

Data are presented as mean values ± standard error of the mean (S.E.M.). Since increases in circulating glucagon did not plateau during hypoglycaemia, data are reported as the concentration 40 min after the establishment of euglycemia and hypoglycaemia (i.e., 80 and 160 min after the start of the clamps) rather than as the area under the curve (AUC). These time points were chosen based on the previous DEPTH trial, which indicated that 40 min is the minimum time needed for glucagon levels to reach a plateau during a hypoglycaemic clamp. Extending the duration beyond these points is unlikely to yield additional information and would unnecessarily increase participant burden and discomfort. Qualitatively, glucagon responses to hypoglycaemia are similar when data are expressed as AUC, but the relative effect is reduced by about 50%. Statistical significance was tested using Student's or Welch's t-test, Wilcoxon signed-rank test, or Wilcoxon matched-pairs signed-rank test, as well as ANOVA followed by Tukey's post-hoc test for multiple comparisons. The data were first analysed using Prism (Version 9.5.0, RRID:SCR_002798, GraphPad) and then re-analysed with SAS v9.4. The standard error of two independent samples was calculated using the Practistat software package.^29^ The statistical analysis plan (SAP) is included as an Appendix.

### Role of funding source

The study funder had no involvement in study design, data collection, data analysis, data interpretation, or report writing. The corresponding author had full access to all study data and was responsible for the final decision to submit the report for publication.

## Results

### Experimental in vitro studies using isolated human islets from donors with T1D

**Fig. 2:**
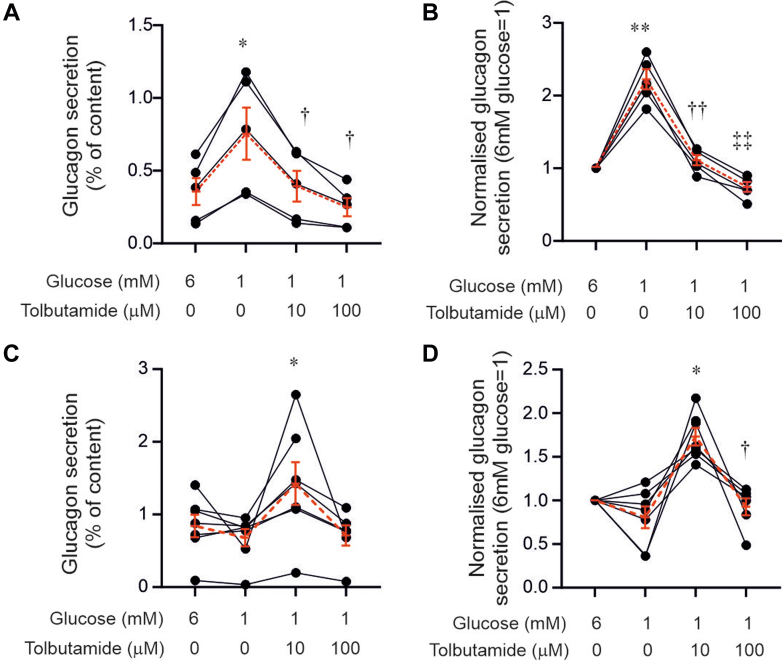
**Tolbutamide restores glucagon secretion at low glucose in T1D islets.** (A) Glucagon secretion in islets from healthy (non-diabetic) donors at 6 and 1 mM glucose with 10 or 100 μM tolbutamide as indicated. ∗p = 0.043 vs 6 mM glucose; †p = 0.028 vs 1 mM glucose. (B) Relative effects of varying glucose and application of tolbutamide as in (A). ∗∗p = 0.0033 vs 6 mM glucose; ††p = 0.0016 vs 1 mM glucose; ‡‡p = 0.0079 vs 10 μM tolbutamide. Responses in different donors are connected by lines. Responses are normalised to glucagon secretion at 6 mM glucose (=1). (C–D) As in (A–B) but using islets from 7 donors with T1D. Significance in (C): ∗p = 0.0292 vs 1 mM glucose. Significance in (D): ∗p = 0.017 vs 1 mM glucose; †p = 0.0353 vs 10 μM tolbutamide. One-way ANOVA followed by Tukey's. Red lines. The bars and error bars represent mean values ± S.E.M under the respective experimental conditions (connected by dashed red lines).

T1D impairs glucagon secretion in response to hypoglycaemia.^3^ We compared how glucose regulates glucagon secretion in isolated human islets in vitro; we found that lowering extracellular glucose from 6 to 1 mM (to mimic severe hypoglycaemia in vivo) did not increase glucagon secretion in islets from donors with T1D, unlike what we observe in islets from control donors without diabetes (Fig. 2). In control islets, the K_ATP_ channel blocker tolbutamide at 10 μM (close to its K_i_ for K_ATP_ channel inhibition, about 7 μM)^30^ reversed the stimulatory effect of glucose lowering (Fig. 2A–B). Glucagon secretion in the presence of 100 μM tolbutamide and 1 mM glucose was more strongly inhibited than at 6 mM glucose (p = 0.0488). However, in islets from donors with T1D, 10 μM tolbutamide consistently (7/7 preparations; 4 males and 3 females) increased glucagon secretion at 1 mM glucose (Fig. 2C–D). Notably, a tenfold higher dose of tolbutamide (100 μM) did not stimulate glucagon secretion, and the rate of release was lower than with 10 μM tolbutamide. Fractional glucagon release (secretion normalised to islet glucagon content) at 6 mM glucose was higher in T1D than in control islets (Fig. 2A–C; p = 0.0017, Welch's t-test). Insulin secretion was undetectable in islets from donors with T1D. Consistent with our previous findings,^8^ islets from donors with T1D hypersecrete somatostatin at 1 mM glucose (0.3–0.7% vs. <0.2% of contents/h in control islets), and this secretion is further stimulated by tolbutamide (Supplementary Figure S4).

### Clinical studies: LEGEND-D trial

Collectively, the in vitro studies suggest that the loss of counterregulatory glucagon secretion in T1D results from defect(s) intrinsic to the alpha-cells and leads to increased K_ATP_ channel activity. They also indicate that properly dosed sulfonylureas may correct the secretion defect. To test this, we conducted the LEGEND-D trial. Given published reports that SGLT2 inhibitors increase glucagon secretion both in vivo and in vitro,^17^^,^^31^^,^^32^ we also tested whether a single dose of dapagliflozin would restore glucagon secretion during hypoglycaemia in people with T1D.

### Plasma glucagon is dysregulated in T1D

**Fig. 3:**
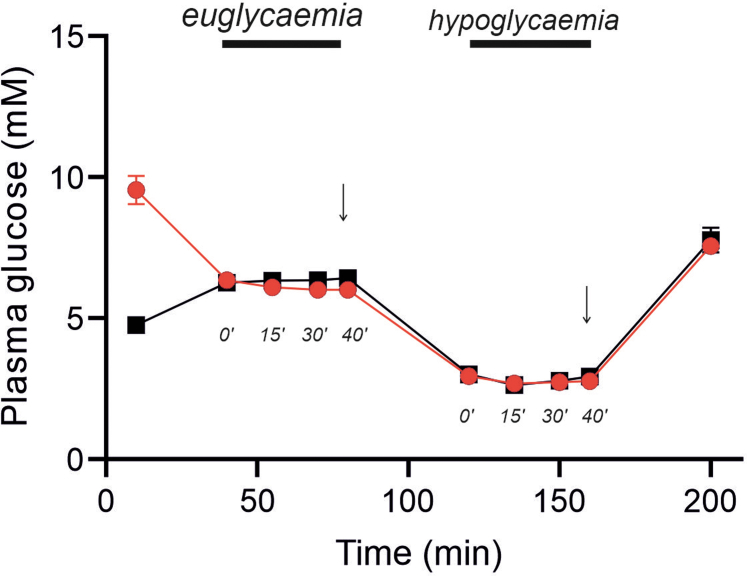
**Hyperinsulinaemic-euglycaemic-hypoglycaemic clamps.** Changes in plasma glucose in healthy individuals (black; n = 6) and participants with T1D (red; n = 59 clamps) are shown, along with blood sampling times. Times (in minutes) after establishing eu- and hypoglycaemic conditions (e.g., 0′, 15′) are indicated for data presentation. Arrows mark the time points (40′) used for statistical analyses and comparisons under euglycaemic and hypoglycaemic states. Data are presented as mean values ± S.E.M.

**Fig. 4:**
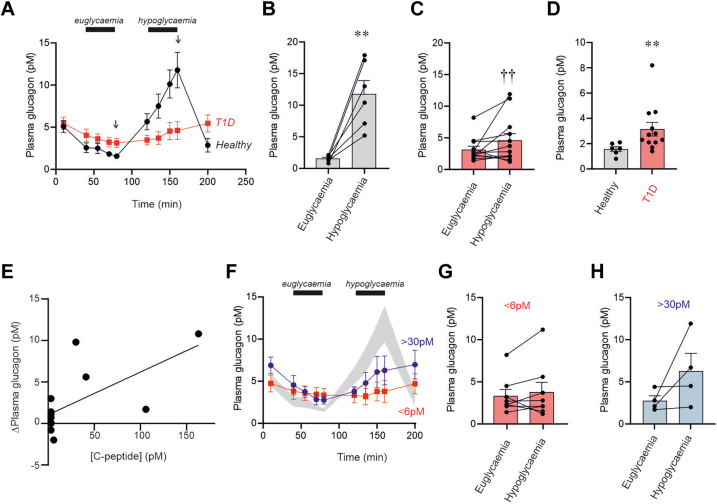
**Impaired regulation of circulating glucagon in T1D.** (A) Changes in plasma glucagon in response to euglycaemia and hypoglycaemia (as indicated) in healthy (black) and T1D (red) participants. (B–C) Plasma glucagon under euglycaemic and hypoglycaemic conditions in healthy (B) and T1D (C) participants. Data are taken at t = 40 min during euglycaemia and hypoglycaemia as indicated by arrows in (A). ∗p = 0.034 vs. euglycaemia in B and ††p = 0.0065 vs. hypoglycaemia in (B). (D) Plasma glucagon in healthy and T1D participants during euglycemia. ∗∗p = 0.0044 vs. healthy participants. (E) Relationship between circulating C-peptide levels ([C-peptide]) and net hypoglycaemia-induced elevation of plasma glucagon (ΔPlasma glucagon). The line is a least-squares fit of the data points to the equation ΔPlasma glucagon = 0.05155∗[C-peptide] + 1.04 (r^2^= 0.4271; p = 0.0212). (F) Plasma glucagon in T1D participants with plasma C peptide concentrations of 6 pM (red) and 30 pM (blue). The grey area represents the range of responses to hypoglycaemia in healthy participants (±SEM; data from [A]). (G–H) Similar to (B–C) but comparing responses in T1D participants with low or high C peptide levels (as indicated). Statistical significance in B–D and G–H was calculated using the Wilcoxon matched-pairs signed-rank test.

We first measured plasma glucagon in the absence of glibenclamide or dapagliflozin under euglycaemic (6–6.4 mM) and hypoglycaemic (2.8–3 mM) conditions in healthy controls and in participants with T1D (Fig. 3). In healthy participants, plasma glucagon under euglycemic conditions was low (1.6 ± 0.2 pM) and increased by 718 ± 93% (n = 6) after 40 min of hypoglycaemia (Fig. 4A–B). In people with T1D, the response to hypoglycaemia was limited to 53 ± 32% (n = 12; p = 0.0178 vs the stimulatory effect in healthy participants: Student's t-test) (Fig. 4C). Notably, plasma glucagon was higher in T1D than in healthy participants under euglycemic conditions (Fig. 4A–D). Somatostatin averaged approximately 100 pM in both healthy participants and those with T1D and was unaffected by changes in plasma glucose (Supplementary Figure S5).

We recognise that all participants with T1D were men. In the control group, there were 4 men and two women. We compared responses in the control group between men and women, and responses were similar, with plasma glucagon increasing from 1.5 to 2 pM under euglycaemic conditions to over 10 pM 40 min after hypoglycaemia induction in both sexes (Supplementary Figure S6).

It has been reported that residual beta-cell function (as reported by detectable serum C-peptide) offers some protection against hypoglycaemia.^25^ Indeed, the net increase in plasma glucagon during hypoglycaemia correlated linearly with C-peptide (r^2^= 0.4274; p = 0.0212), with glucagon increasing 5 pM for every 100 pM of C-peptide (Fig. 4E). In Fig. 4F–H, we compared plasma glucagon levels under euglycemic and hypoglycaemic conditions in participants with T1D with C-peptide levels ≥30 pM or ≤6 pM. In low C-peptide participants the response to hypoglycaemia averaged 10 ± 15% (n = 8; p < 0.0001 compared with hypoglycaemia in healthy individuals) (Fig. 4E–F). In participants whose C-peptide levels were ≥30 pM, the corresponding value was 141 ± 79% (n = 4), with 2 of the 4 participants showing a healthy response to hypoglycaemia (Fig. 4G).

### Glibenclamide raises plasma glucagon in T1D

We next tested the capacity of increasing doses of glibenclamide (0.3–3 mg/day) to restore plasma glucagon levels during hypoglycaemia in T1D. The in vitro experiments suggested that tolbutamide at concentrations near its K_i_ for inhibition of K_ATP_ channels stimulates glucagon secretion in islets from donors with T1D. However, the equivalent dose of glibenclamide required to achieve partial closure in humans is unknown. Therefore, we conducted the trial using multiple glibenclamide dosages.

**Fig. 5:**
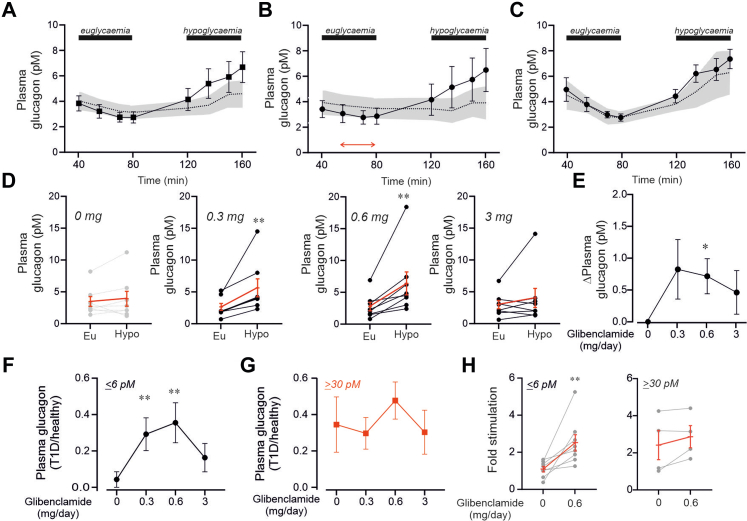
**Increased circulating glucagon in participants with T1D by low-dose glibenclamide.** (A–C) Plasma glucagon levels under eu- and hypoglycaemic conditions in participants with T1D (n = 12: A), in those with T1D and C-peptide ≤6 pM (n = 8: B), and in those with T1D with C-peptide ≥30 pM (n = 4: C), all treated with 0.6 mg/day glibenclamide. The dotted line and grey area indicate plasma glucagon levels measured in the same participants without glibenclamide. (D) Plasma glucagon during hypoglycaemia in T1D participants with C-peptide 6 pM under eu- and hypoglycaemic conditions with treatments of 0 (same as in Fig. 4F), 0.3, 0.6, and 3 mg/day glibenclamide, as indicated. ∗∗p = 0.0078 vs euglycaemia within the same treatment group (Wilcoxon matched-pairs signed-rank test). Red lines and error bars show mean values ± S.E.M. of individual experiments (n = 8). (E) Net effects of glibenclamide dosage on plasma glucagon under euglycaemic conditions in T1D participants with C-peptide ≤6 pM ∗p = 0.039 vs no glibenclamide (Wilcoxon matched-pairs signed-rank test). Baseline was the average of data points indicated by the red arrow in B. (F–G) Effects of increasing glibenclamide on plasma glucagon during hypoglycaemia in participants with T1D with ≤6 pM (F) and ≥30 pM C-peptide (G). Data expressed as a fraction of the response in healthy individuals not receiving. In F ∗∗p = 0.0030 (for 0.3 mg/day) or 0.0015 (0.6 mg/day) glibenclamide (ANOVA with Friedman test). No statistically significant effects in G. (H) Fold elevation of plasma glucagon in response to hypoglycemia in participants with C-peptide levels ≤6 pM (left) or ≥30 pM (right) with or without treatment with 0.6 mg/day glibenclamide as indicated. ∗∗p = 0.0078 vs no glibenclamide (Wilcoxon matched-pairs signed rank test).

Fig. 5 compares the changes in plasma glucagon during the hyperinsulinaemic euglycaemic and hypoglycaemic clamps in all participants with T1D (Fig. 5A) and in the subgroups with plasma C-peptide levels ≤6 pM (Fig. 5B) and ≥30 pM (Fig. 5C) before (grey area) and after (black symbols) treatment with 0.6 mg/day glibenclamide (chosen for illustrative purposes because this is the dosage with the strongest effect; see below). The sulfonylurea had a particularly strong effect in participants lacking C-peptide. In these individuals, glibenclamide increased the counterregulatory rise in plasma glucagon by 3.0 ± 0.8 pM (n = 8; p = 0.0156 by Wilcoxon matched-pairs signed rank test), an effect limited to 1.1 ± 1.2 pM (n = 4; p = 0.625) in those with C-peptide. Overall, the effect of glibenclamide across all participants averaged 2.3 ± 0.7 pM (n = 12; p = 0.0088).

Fig. 5D compares plasma glucagon 40 min after the induction of euglycaemia and hypoglycaemia in the absence and presence of increasing glibenclamide doses (0.3–3 mg/day). Glibenclamide at 0.3 and 0.6 mg/day increased glucagon by 125 ± 23% (p = 0.0030) and 152 ± 44% (p = 0.0110), respectively (ANOVA followed by Friedman test)—much higher than the 10 ± 15% (see above) observed in these participants without glibenclamide. However, at 3 mg/day, the effect was limited to 34 ± 23% and was not statistically significant (p > 0.9999).

Additionally, glibenclamide (0.6 mg/day) reduced plasma glucagon under euglycaemic conditions (Fig. 5E).

Fig. 5F–G illustrates the dose-dependent effects of glibenclamide on plasma glucagon in participants with T1D who either lack (C-peptide ≤6 pM) or retain some beta-cell function (C-peptide ≥30 pM). Data are shown as a fraction of the responses seen in healthy individuals. The response in participants with T1D without C-peptide exhibits a bell-shaped dependence on glibenclamide dose, increasing from 4% without the sulfonylurea to a peak of 36% at 0.6 mg/day (Fig. 5F). In the small subgroup of participants with T1D who retained some C-peptide (n = 4), glibenclamide did not produce any statistically significant effects beyond those caused by C-peptide alone (34% of plasma glucagon in healthy individuals) (Fig. 5G).

Results were similar when the model was adjusted for dose sequence, age, BMI, blood pressure, and heart rate (Supplementary Table S2).

We addressed the inter-individual variability by calculating the fold increase in plasma glucagon with and without glibenclamide (0.6 mg/day). In the low C-peptide group, an improved response (20–720%) to hypoglycaemia was observed in every participant and exceeded 50% in 6 of the 8 participants. In the high-C-peptide group, an improvement greater than 50% was observed in only one participant (Fig. 5H).

### Glibenclamide influences glucose infusion rate required to maintain eu- and hypoglycaemia

**Fig. 6:**
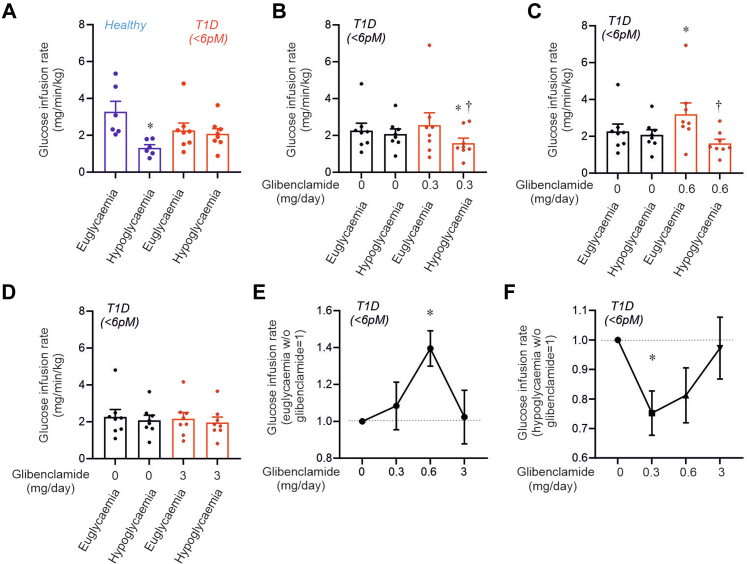
**Glibenclamide restores hypoglycaemia-induced glucose production in participants with T1D.** (A) Glucose infusion rates in healthy controls and participants with T1D lacking C-peptide during euglycemia and hypoglycaemia, as indicated. ∗p = 0.0312 vs euglycemia in healthy individuals. (B–D) Dosage-dependent (0.3–3 mg/day) glibenclamide-induced reduction in glucose infusion requirements during euglycemia and hypoglycaemia in participants with T1D without C-peptide. Statistical significance in (B): ∗p = 0.0234 vs hypoglycaemia without glibenclamide; †p = 0.0391 vs euglycemia with glibenclamide (0.3 mg/day). Statistical significance in (C): ∗p = 0.0156 vs euglycemia without glibenclamide; †p = 0.0078 vs euglycemia with glibenclamide (0.6 mg/day). (E–F) Effects of increasing glibenclamide doses on glucose infusion rate under eu- (E) and hypoglycaemic (F) conditions. Responses are normalised to glucose infusion rates without glibenclamide under each respective condition. ∗p = 0.0247 in (E) and ∗p = 0.0234 in (F). Dotted lines indicate normalised glucose infusion rates under eu- and hypoglycaemic conditions, respectively. Statistical significance was determined using the Wilcoxon matched-pairs signed-rank test.

We measured the glucose infusion rate during euglycaemia and hypoglycaemia. Notably, a decrease in the infusion rate indicates it is balanced by endogenous (hepatic) glucose production. In healthy individuals, hypoglycaemia reduced glucose infusion rate by 60%, whereas in people with T1D lacking C-peptide (the group with the particularly strong effect on plasma glucagon), no such reduction was observed (Fig. 6A). In these participants, the glucose infusion rate under euglycaemic conditions also tended to be lower than in healthy individuals, but this difference was not statistically significant (p = 0.1775 by Welch's t-test). These defects were reversed by glibenclamide treatment: 0.3 mg/day reduced glucose infusion rate during hypoglycaemia, while 0.6 mg/day both increased it under euglycaemia and decreased it during hypoglycaemia (Fig. 6B–C). Indeed, at 0.6 mg/day, glucose infusion rates in participants with T1D overlapped with those in healthy participants. No effects on glucose infusion rate were observed with 3 mg/day glibenclamide (Fig. 6D). The dose-dependent stimulatory and inhibitory effects under eu- and hypoglycaemic conditions are summarised in Fig. 6E–F, illustrating the U- and bell-shaped relationships. We attribute these reciprocal effects of 0.3–0.6 mg/day glibenclamide on glucose infusion rates to reductions and increases in circulating glucagon induced by the sulfonylurea under eu- and hypoglycaemic conditions, respectively (Fig. 5). The statistical significance remained unchanged when including data from all 12 participants, but the relative effects were weaker as glibenclamide had no stimulatory or inhibitory effects on glucose infusion rate in participants with C-peptide levels below 30 pM (Supplementary Figure S7A).

### Dapagliflozin increases counterregulatory glucagon and modulates glucose infusion rate

**Fig. 7:**
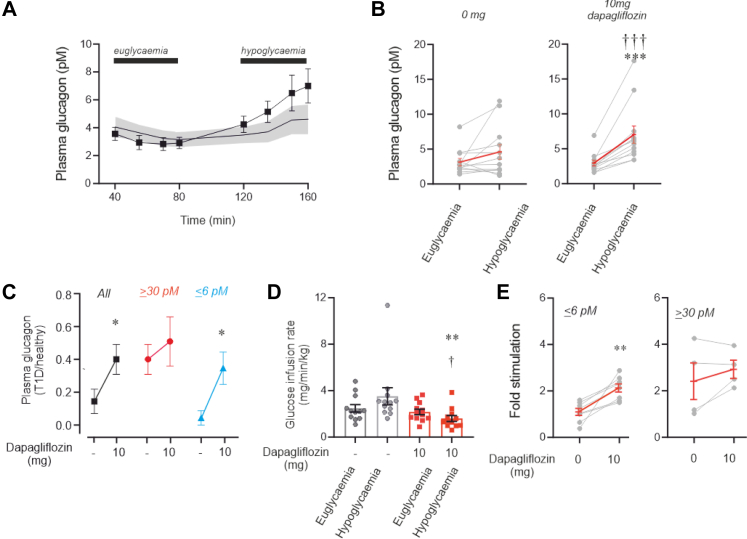
**Dapagliflozin restores counterregulatory plasma glucagon and glucose production.** (A) Changes in plasma glucagon in response to euglycemia and hypoglycemia in participants with T1D, both with and without C-peptide (≤6 pM), under basal conditions (grey area; ±S.E.M.) and after acute administration of dapagliflozin (10 mg; black symbols/line). (B) Plasma glucagon under euglycemic and hypoglycaemic conditions in participants without C-peptide, with (right) or without (left) dapagliflozin treatment, as indicated. Baseline values (left) are the same as in Fig. 3F and are shown here for comparison. ∗∗p = 0.0078 (Wilcoxon matched-pairs signed-rank test). (C) Plasma glucagon in participants with T1D with C-peptide levels ≤6 pM (n = 8) or ≥30 pM (n = 4) under hypoglycaemic conditions in the basal state (no treatment) and after treatment with dapagliflozin. Responses have been normalised to response in healthy individuals not receiving any medication. ∗∗p = 0.0078 vs euglycaemia with dapagliflozin (Wilcoxon test). (D) Glucose infusion rate in T1D participants with low C-peptide (≤6 pM), measured at baseline and after dapagliflozin treatment. ∗∗p = 0.0078 vs no dapagliflozin (Wilcoxon signed-rank test).(E) Fold elevation of plasma glucagon in response to hypoglycemia in participants with C-peptide levels ≤6 pM (left) or ≥30 pM (right) with or without treatment with 10 mg dapagliflozin as indicated. ∗∗p = 0.0078 vs no glibenclamide (Wilcoxon matched-pairs signed rank test). In (A, B and D), red lines and error bars represent mean values ± S.E.M. of individual experiments (n = 8).

We also examined the acute effects of dapagliflozin on plasma glucagon. We observed that a single dose of dapagliflozin, administered 2 h before the clamps, increased plasma glucagon during hypoglycaemia in participants with T1D lacking C-peptide (Fig. 7A–C). In these individuals, the SGLT2 inhibitor increased the counterregulatory rise in plasma glucagon by 3.1 ± 0.8 pM (n = 8; p = 0.0078 by Wilcoxon matched-pairs signed-rank test), whereas the effect was limited to 0.9 ± 1.9 pM (n = 4; p > 0.9999) in those with C-peptide. Overall, the effect of dapagliflozin across all participants averaged 2.4 ± 0.8 pM (n = 12; p = 0.022). In the low-C-peptide group, an improved response (48–767%) to hypoglycaemia was observed in every participant. In the high-C-peptide group, the effects of dapagliflozin were less consistent, with glucagon levels reduced in 2 participants (Fig. 7E). Again, including all 12 participants made no difference statistically, but the relative effect was weaker. Dapagliflozin decreased the glucose infusion rate during hypoglycaemia but had no effect during euglycemia (Fig. 7E), consistent with the effects on plasma glucagon under each condition. Dapagliflozin showed no additive effect on either glucagon release (Fig. 7C and Supplementary Figure S8) or glucose infusion rate (Supplementary Figure S7B) in participants who still produced some insulin, indicated by detectable C-peptide.

### Glibenclamide improves glycaemic control in T1D

**Fig. 8:**
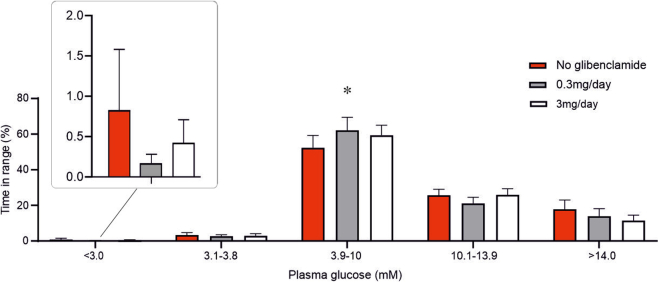
**Improved glucose control (‘time-in-range’) in patients with T1D treated with low-dose glibenclamide.** Fraction of time spent in different plasma glucose ranges before and after treatment with 0.3 mg/day and 3 mg/day glibenclamide. ∗p = 0.033 versus baseline (no glibenclamide). Inset: Data for time spent below 3 mM glucose on an expanded vertical axis. All participants included regardless of C-peptide levels.

Finally, we assessed glycaemic control by measuring time-in-range (TIR; i.e., 3.9–10 mM) using continuous glucose monitoring in all 12 participants with T1D with and without glibenclamide. We observed an increase in TIR from 52% to 62% with 0.3 mg/day of glibenclamide, mainly due to a reduction in hyperglycaemia (Fig. 8), which approached statistical significance (p = 0.078). This improvement was not seen at higher doses of 0.6 or 3 mg/day. Although a trend towards reduced occurrence of hypoglycaemia with 0.3 mg/day glibenclamide was observed, this effect did not reach statistical significance (Fig. 8, inset). Detailed analysis showed that 90% of the improvement at <3 mM glucose was attributable to a single participant.

### Adverse events

Only one adverse event was reported: mild dysphagia while taking the maximum dose of 3 mg/day glibenclamide. This event was classified as an adverse reaction (AR) because it occurred at the start of the 3 mg/day glibenclamide protocol and resolved after discontinuing glibenclamide. The participant had no issues at lower doses. They continued taking the medication after symptoms appeared, and the dysphagia fully resolved during follow-up. This event was categorised as an AR due to the potential causal link with the treatment. It was reported to MRHA and documented in the trial and medical records. No other adverse events were reported: hypoglycaemia requiring third-party assistance or DKA were not observed during the trial. Ketone levels measured after dapagliflozin treatment remained well below 0.6 mM in all participants.

## Discussion

Both T1D and T2D are bihormonal disorders characterised by abnormalities in insulin and glucagon secretion. In T1D, glucagon secretion is impaired in two key ways: there is excess circulating glucagon at normal blood glucose levels, worsening the impact of insulin deficiency during hyperglycaemia, and there is too little circulating glucagon when blood glucose levels drop, increasing the risk of life-threatening hypoglycaemia. In this hypothesis-generating and exploratory study, we now demonstrate that both defects are partially corrected by low-dose glibenclamide. We acknowledge that the observed effects may be influenced by period effects or the number of prior visits, but the randomised trial design militates against this possibility and baseline plasma glucagon remained within ±13% throughout the study.

The physiological regulation of glucagon secretion is complex. It was previously suggested that high intra-islet insulin levels suppress glucagon secretion and that the stimulation of insulin secretion mediates the inhibitory effect of high glucose on glucagon secretion; however, this is questionable given that glucose exerts its maximum inhibitory effect on glucagon secretion from pancreatic islets at concentrations that have little or no stimulatory effect on insulin secretion.^24^^,^^33^ It is also notable that in vivo glucagon secretion is triggered by insulin-induced hypoglycaemia when blood insulin levels are elevated to supraphysiological levels; this is the opposite of what would be expected if insulin were the primary regulator of glucagon release.^3^ Indeed, ablating insulin receptors on alpha-cells only slightly impairs the increase in circulating glucagon in response to hypoglycaemia.^34^ Thus, a drop in plasma glucose is more likely to trigger glucagon release than a decrease in insulin within the islet. Physiologically, there is a glucose concentration-dependent hierarchy of counterregulatory responses: First, insulin secretion is switched off, and then, at lower glucose concentrations, glucagon secretion is stimulated. If glucagon secretion fails (or if glucose continues to fall), adrenaline secretion increases.^35^ The similarity of responses to sulfonylureas in vitro (where innervation and blood flow are cut off) and in vivo, along with the bell-shaped relationship between dose and glucagon secretion, supports the idea that sulfonylureas act directly on the pancreatic islets rather than through a different (indirect) mechanism.

While our data support a role for K_ATP_- and somatostatin-dependent mechanisms, defective counterregulation is likely to be multifactorial, and we cannot exclude the involvement of autonomic, neuronal, paracrine, or other systemic mechanisms. We previously discovered that AVP from the posterior pituitary increases circulating glucagon during hypoglycaemia, and this effect is impaired in T1D.^36^ Of particular relevance for this study is that factors that inhibit somatostatin secretion and action, including free fatty acids^37^^,^^38^ or somatostatin antagonists,^39^ also stimulates glucagon secretion.

Previous experimental studies have suggested that glucagon secretion is controlled by a mosaic of K_ATP_ and somatostatin-sensitive G protein-gated inwardly rectifying potassium (GIRK) channels.^40^ K_ATP_ and GIRK channel activities might increase due to decreased mitochondrial ATP production^41^ and stimulation of somatostatin release,^8^ respectively. Glucagon secretion is suppressed when the combined activity of these two types of channels becomes too high. The observation that low—but not high—concentrations of glibenclamide increase plasma glucagon supports the idea that there is a bell-shaped relationship between K^+^ channel activity and glucagon release in vivo, a relationship that has previously been documented in isolated healthy islets.^24^ Somatostatin secretion at both low and high concentrations of tolbutamide was similar in isolated islets, indicating that the different effects on glucagon secretion are unlikely to be caused by stronger paracrine inhibition at higher concentrations. Somatostatin secretion is significantly increased in isolated islets from donors with T1D.^8^

We have previously shown that dapagliflozin inhibits somatostatin secretion in human islets under experimental conditions that mimic hyperinsulinaemic-hypoglycaemic clamp studies (4 mM glucose and ≥300 pM insulin^31^). It is therefore possible that dapagliflozin increases circulating glucagon levels by decreasing intra-islet somatostatin release. Alternatively, dapagliflozin may exert a direct effect on the glucagon-secreting alpha-cells.^32^^,^^42^ In support of the involvement of somatostatin, the loss of counterregulatory increase in circulating glucagon in T1D is partially corrected by the somatostatin receptor 2 (SSTR2) antagonist CYN154806,^8^ and dapagliflozin has little additive stimulatory effect on glucagon secretion when added in the presence of the SSTR2 antagonist.^43^ A direct effect of dapagliflozin in the islets is seemingly surprising given the low expression of *SLC5A2* (which encodes SGLT2) in human islet cells. However, functional measurements showing dapagliflozin-sensitive increases in cytosolic Na^+^ upon application of the non-metabolisable SGLT-specific substrate methyl-α-d-glucopyranoside (αMDG)^43^ and the presence of SGLT2 in human delta-cells was confirmed by immunocytochemistry.^31^ We note that the effects of dapagliflozin on plasma glucagon during the clamps were detected within 2–3 h of administration, which coincides with the time of peak plasma concentration. The rapid kinetics favours a direct action rather than long-term metabolic adaptations.^44^^,^^45^ Renal glucose loss can also be excluded as an underlying factor, as the effects were observed even when plasma glucose was clamped. The finding that dapagliflozin reduced the glucose infusion requirement is also not easily reconciled with the idea that the effects on plasma glucagon are mediated by increased glucose excretion.

Although elevated plasma glucagon cannot be equated with a reduced hypoglycaemia risk and should therefore be regarded as a surrogate marker, the capacity of glibenclamide and dapagliflozin may be clinically relevant as it stimulates endogenous (hepatic) glucose production, as evidenced by the reduced glucose infusion rate needed to maintain plasma glucose. In the presence of dapagliflozin or low-dose glibenclamide, glucagon levels during hypoglycaemia are approximately 35% of those observed in healthy individuals and are comparable to the modest counterregulatory response that persists in people with T1D in whom residual beta-cell function persists, as evidenced by circulating C-peptide levels ≥30 pM. This subgroup is admittedly very small (n = 4), warranting caution when interpreting these data, but this notion is supported by the protective effect of circulating C-peptide reported in a much larger study.^25^ The underlying mechanism has been an enigma, but we recently proposed, based on work on human islets from organ donors with T1D, that it reflects the capacity of beta-cells to function as an electrical brake on the delta-cells.^8^ Since there are more beta-than delta-cells in pancreatic islets, 80–90% of beta-cells can be destroyed while delta-cells remain in electrical contact with at least one beta-cell. It is only when all beta-cells are gone that their restraining effect on delta-cell electrical activity and somatostatin secretion is lost. When this happens, somatostatin is oversecreted, leading to paracrine suppression of glucagon.^8^ Our observation of a linear correlation between circulating C-peptide and the net hypoglycaemia-induced elevation in plasma glucagon is consistent with this concept. Our results with dapagliflozin appear to conflict with those of Pettus and colleagues, who did not observe an improved glucagon response to hypoglycaemia in people with T1D.^46^ However, the participants in this study included those with higher circulating C-peptide (cut-off: 0.7 ng/ml, equivalent to 230 pM), and they retained a small counterregulatory response, as we observed in participants with C-peptide levels above 30 pM.

Glibenclamide (tablets) is available in dosages of 1.75–5 mg, which approach or exceed levels where glibenclamide loses its effectiveness. However, it is also available as an oral suspension (Amglidia) that has marketing authorisation in Europe and the UK for neonatal use in diabetes,^47^ thus enabling accurate dosage. Dapagliflozin previously held marketing authorisation for glycaemic control in T1D and was recommended by the National Institute for Health and Care Excellence (NICE).^48^ However, the manufacturer decided to withdraw the licence.^49^ Of note, in a meta-analysis of trials involving people with T1D comparing dapagliflozin plus insulin to insulin alone, there were no increases in hypoglycaemia despite improvements in HbA1c.^50^

We note that we did not recruit the planned number of participants, but that the changes induced by glibenclamide and dapagliflozin were larger than anticipated. We further acknowledge that all 12 participants with T1D were males. The predominance of males in diabetes trials is well known.^51^ In our trial, participants were enrolled on a ‘first-come, first-served’ basis. As this was an early-phase study involving a prolonged and intensive protocol, the exclusion criteria included pregnancy, breastfeeding, and women of childbearing potential who were not using adequate contraception. The requirement for strict contraceptive measures throughout the study period may have reduced willingness among some women to participate. We are unaware of any evidence that females have a lower incidence of hypoglycaemia^52^ and we did not observe any difference in response to hypoglycaemia between males and females among the healthy controls. In the in vitro studies, low concentrations of sulfonylureas were equally effective at stimulating glucagon secretion in islets from male and female donors. Nonetheless, it is important to verify that any benefits of glibenclamide on glucagon secretion and blood glucose regulation also apply to women.

In conclusion, medications traditionally used to manage blood sugar in type 2 diabetes, specifically glibenclamide and dapagliflozin, appear to improve plasma glucagon levels and time-in-range for blood glucose in people with T1D who do not produce endogenous insulin. This treatment may help correct both excessive circulating glucagon during normal blood sugar levels and insufficient glucagon during hypoglycaemia. Indeed, we found that 0.3 mg/day of glibenclamide increased TIR (3.9–10 mM), which may reflect a reduction in plasma glucagon under normal blood sugar conditions, leading to decreased blood sugar variability.^53^ Our pilot study was not designed to detect a reduced incidence of severe hypoglycaemia, and prior hypoglycaemia was, in fact, an exclusion criterion. We propose that glibenclamide or dapagliflozin should be considered as an adjunct to insulin in T1D, and support translating these findings into larger clinical trials that assess the effects of these treatments on glycaemic control, hypoglycaemia incidence, ketoacidosis, and health-related quality of life.

## Contributors

Rui Gao, Ioannis Spiliotis: conceptualisation, data curation, investigation, formal analysis, methodology; Haiqiang Dou, Thomas Hill, Lakshmi Kothegala, Caroline Miranda: investigation, formal analysis, methodology; Nkemjika Abiakam: clinical trial management; Ruth Coleman, statistical analyses; Sarah White, participant recruitment, allocation of treatments and procedures; Amanda Adler: methodology, project administration, manuscript editing; Patrik Rorsman: conceptualisation, resources, supervision, funding acquisition, project administration, manuscript writing. Rui Gao and Ioannis Spiliotis have accessed and verified the underlying data. All authors approved the final version of the manuscript.

## Data sharing statement

Upon publication, de-identified participant data, the study protocol, and the statistical analysis plan will be deposited in the Oxford Research Archive (http://ora.ox.ac.uk/) with a registered DOI. Data will be retained for 5 years. Mediated access will be granted to qualified researchers for the purposes of academic research and independent verification upon reasonable request to the corresponding author, subject to a signed data access agreement.

## Declaration of interests

We declare no competing interests.

## Appendix A Supplementary data

Supplementary Tables and Figures

Statistical Analysis
