# Supplementary material for: Partial restoration of counterregulatory circulating glucagon by dapagliflozin and low-dose glibenclamide in men with type 1 diabetes: in vitro studies and randomised clinical crossover trial
Source: eBioMedicine. 2026 May 19;128:106298. doi: 10.1016/j.ebiom.2026.106298 (PMC13213787; doi:10.1016/j.ebiom.2026.106298)
Supplement: Supplementary Tables and Figures [file mmc1.docx]

**Supplementary material**

Partial restoration of counterregulatory circulating glucagon by dapagliflozin and low-dose glibenclamide in men with type 1 diabetes:

*in vitro* studies and randomised clinical crossover trial

Rui Gao, Ioannis Spiliotis, Haiqiang Dou, Thomas G. Hill, Lakshmi Kothegala, Caroline Miranda, Nkemjika Abiakam, Ruth L. Coleman, Sarah White, Amanda Adler and Patrik Rorsman

**Supplementary Table 1. Human donor information**

| **Islet  preparation** | **Unique  identifier** | donor age (years) | donor sex (M/F) | donor BMI (kgm^-2^) | Donor  HbA1c (%) | Islet isolation centre | Donor history of T1D |
| --- | --- | --- | --- | --- | --- | --- | --- |
| **1** | **H2532** | 70 | F | 18.7 | 5.5 | Uppsala | T1D |
| **2** | **R429** | 23 | F | 23.9 | 11.8 | Alberta | T1D |
| **3** | **R437** | 38 | F | 38.6 | 10.0 | Alberta | T1D |
| **4** | **H2574** | 44 | M | 25.1 | 5.4 | Uppsala | T1D |
| **5** | **R539** | 57 | M | 28.1 | 8 | Alberta | T1D |
| **6** | **H2685** | 42 | M | 25 | 6.6 | Uppsala | T1D |
| **7** | **H2740** | 45 | M | 23.1 | 6.2 | Uppsala | T1D |
| **7** | **H2530** | 65 | M | 29.4 | 6.1 | Uppsala | No |
| **8** | **H2539** | 75 | M | 23.8 | 5.2 | Uppsala | No |
| **9** | **R396** | 32 | M | 24.8 | 4.5 | Alberta | No |
| **10** | **H2610** | 62 | M | 22.2 | 3.6 | Uppsala | No |
| **11** | **H2613** | 65 | M | 22.3 | 3.8 | Uppsala | No |
| **12** | **H2618** | 80 | M | 27.7 | 4.5 | Uppsala | No |
| **13** | **152** | 58 | M | 26.9 | 5.9 | Uppsala | No |
| **14** | **156** | 64 | M | 28.9 | 6.8 | Uppsala | No |
| **15** | **157** | 69 | M | 25.7 | 5.6 | Uppsala | No |

**LEGEND-D Validation Analyses**

**Table 2a.** Paired t-tests comparing the plasma glucagon concentration (pM) at 40 min during induced hypoglycaemia

| t-test comparator groups | Number of participants | Mean difference (SD) 0mg – X.X mg | p-value |
| --- | --- | --- | --- |
| 0mg vs 0.3 mg | 11 | -1.65 (0.82) | <0.0001 |
| 0mg vs 0.6 mg | 12 | -2.08 (2.50) | 0.015 |
| 0mg vs 3 mg | 12 | 0.37 (1.98) | 0.54 |

**Table 2b.** Linear mixed effects model with random subject effects and adjustment for period and sequence interactions

| Dose | Least Squares Means (95%CI) | p-value |
| --- | --- | --- |
| 0.3 mg | -1.44 (-2.59 - -0.29) | 0.018 |
| 0.6 mg | -2.23 (-3.37 - -1.10) | 0.0008 |
| 3 mg | 0.40 (-0.73 - 1.53) | 0.46 |

**Table 2c.** Comparing sulfonylurea dose

| Dose comparison | Least Squares Mean diff (95%CI) | p-value |
| --- | --- | --- |
| 0.3 mg vs 0.6 mg | 0.81 (-0.34 – 1.96) | 0.16 |
| 0.3 mg vs 3 mg | -1.82 (-2.97 - -0.66) | 0.0038 |
| 0.6 mg vs 3 mg | -2.63 (-3.78 - 1.47) | 0.0001 |

P-values reflect change from baseline

**Table 2d.** Linear mixed effects model as above with additional adjustment for age, BMI, blood pressure (SBP and SBP) and resting heart rate

| Dose | Least Squares Means (95%CI) | p-value |
| --- | --- | --- |
| 0.3 mg | -1.47 (-2.60 - -0.33) | 0.019 |
| 0.6 mg | -2.32 (-3.45 - -1.19) | 0.0022 |
| 3 mg | 0.29 (-0.84 - 1.42) | 0.55 |

**Table ee.** Comparing sulfonylurea dose

| Dose comparison | Least Squares Mean diff (95%CI) | p-value |
| --- | --- | --- |
| 0.3 mg vs 0.6mg | 0.86 (-0.29 – 2.00) | 0.14 |
| 0.3 mg vs 3mg | -1.76 (-2.91 - -0.60) | 0.0049 |
| 0.6 mg vs 3mg | -2.61 (-3.77 - 1.46) | 0.0001 |

P-values reflect change from baseline

**
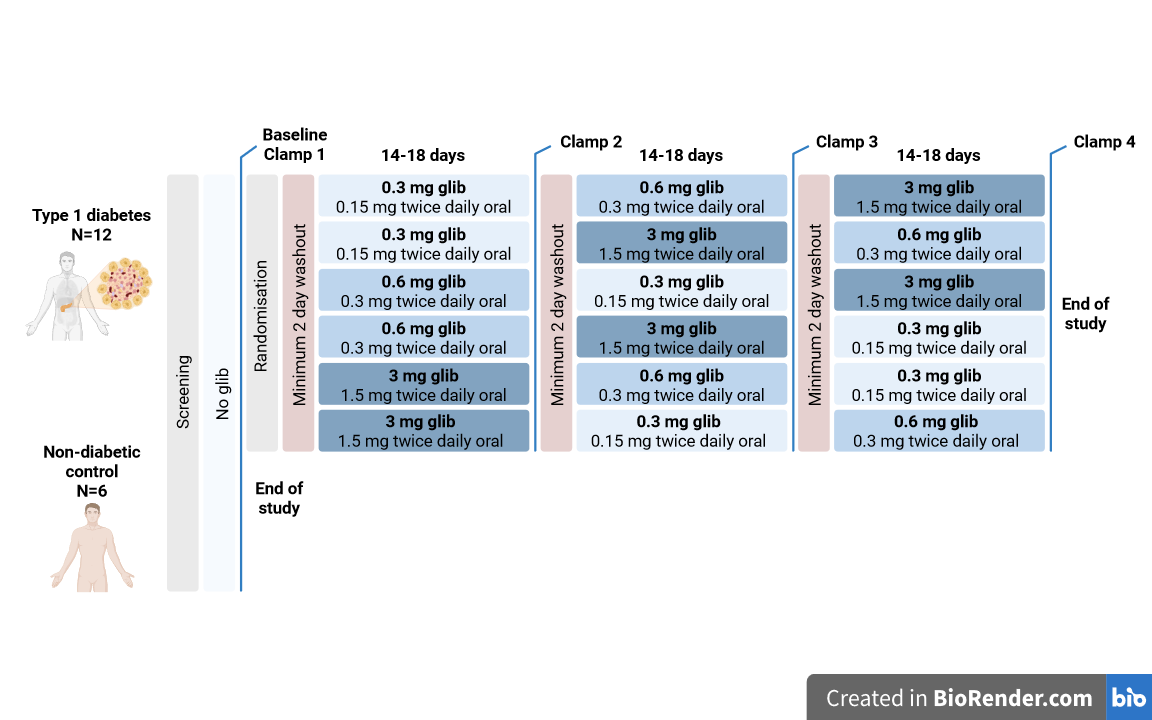
Supplementary Figure 1: Outline of clinical study** (Created by BioRender).

**
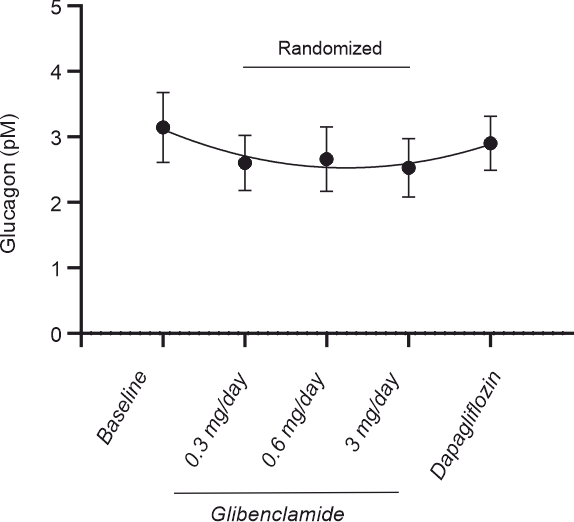
**

**Supplementary Figure 2: Baseline stability.** Plasma glucagon was measured under euglycemia during the five hyperinsulinaemic euglycaemic and hypoglycaemic clamps. The sequence of glibenclamide dosages was randomized.

**
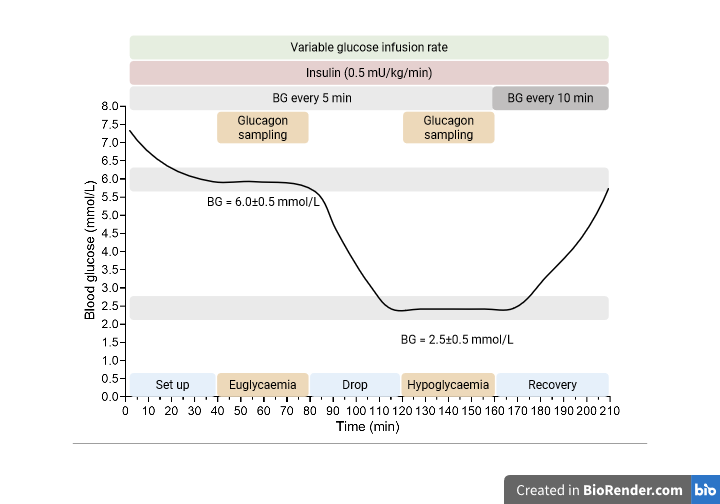

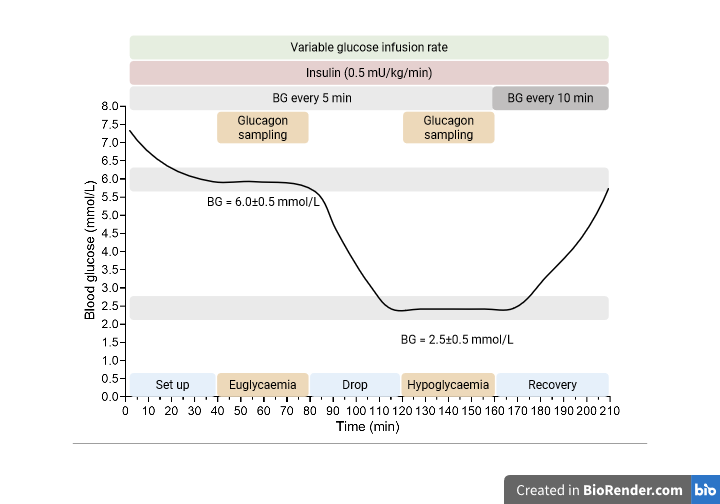

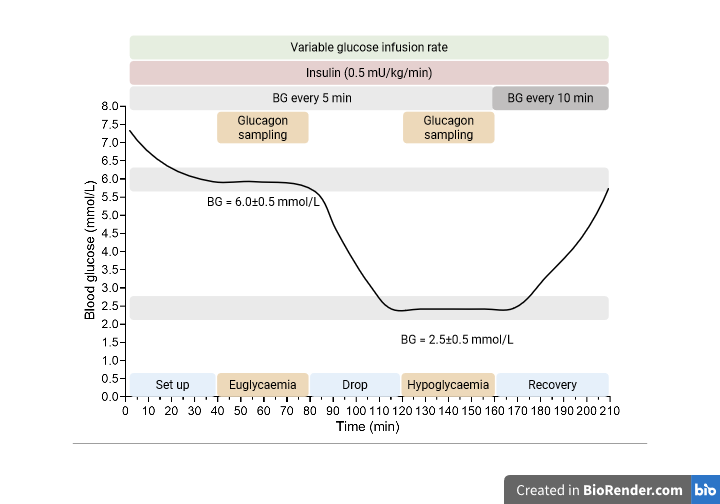
**

**BG: ~6 mM**

**Insulin infusion rate (0.5 mU/kg/min)**

**BG: ~2.5 mM**

**Supplementary Fig. 3: Design of clamp studies** (Created by BioRender). Abbreviation: BG, blood glucose

**
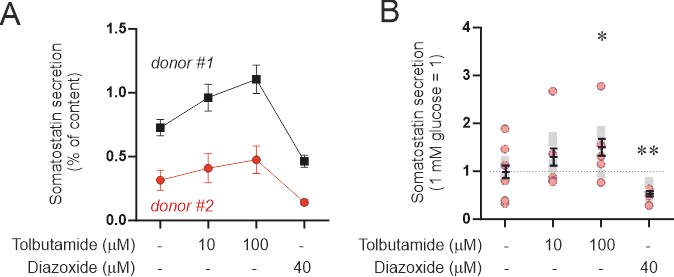
**

**Supplementary Figure 4: Somatostatin secretion in T1D islets.** (A) Somatostatin secretion at 1 mM glucose was measured in the absence and presence of tolbutamide and diazoxide, as indicated. Data were obtained from 2 donors with T1D (donors #1 and 2) and are expressed as fractional release (% of content). Each group of islets (5-6 for each condition and donor) was treated as a separate experiment. (B) Same data as in (A), but secretion in each group has been normalized to the average somatostatin release at 1 mM glucose. Data from each donor are colour-coded. *p=0.0137 and **p<0.0020 vs 1 mM glucose (Wilcoxon signed-rank test). Black lines and error bars represent mean values ± S.E.M. of the individual experiments (n=10-11 from 2 donors). The dotted black line indicates basal secretion at 1 mM glucose.

**
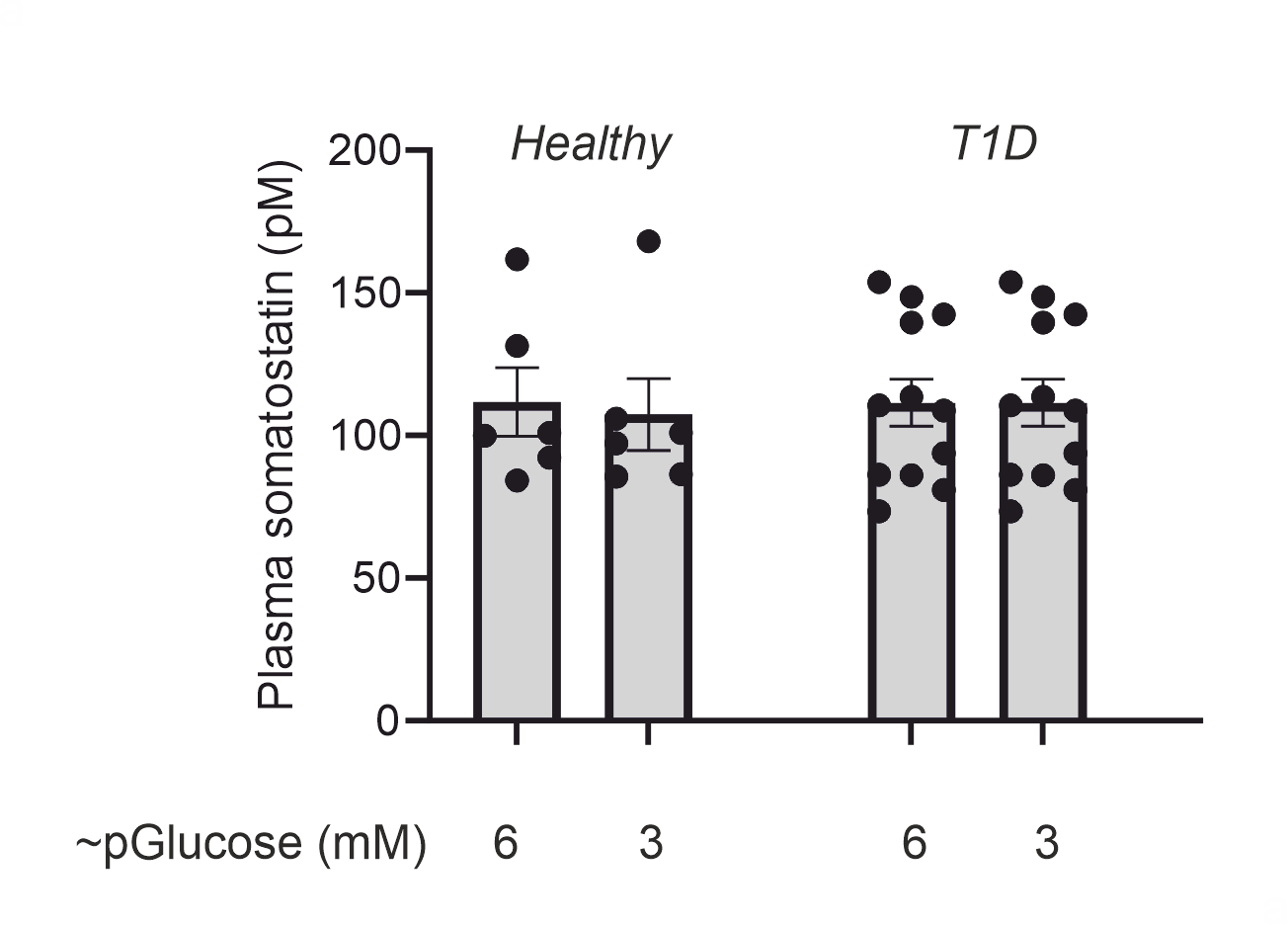
**

**Supplementary Figure 5: Circulating somatostatin in healthy individuals and participants with T1D.** Somatostatin measured under euglycaemic (~6 mM) and hypoglycaemic (~3 mM glucose) conditions in healthy participants and in people with T1D as indicated. Abbreviation: ~pGlucose, approximate plasma glucose concentration (measured in Figure 3).


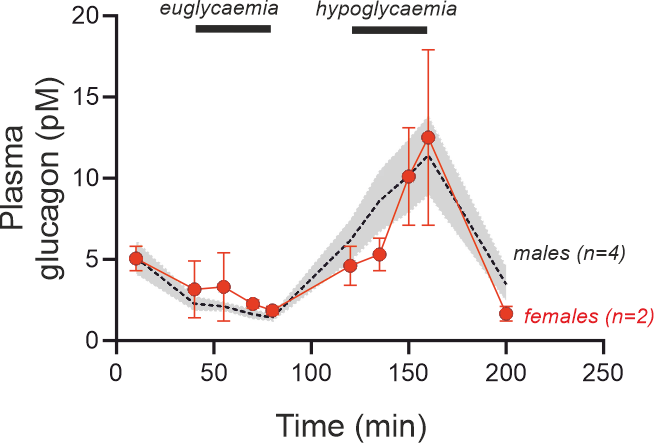


**Supplementary Figure 6: Counter-regulation in men and women.** Changes in plasma glucagon levels in response to euglycemia and hypoglycaemia (as indicated) in healthy males (black shaded area and dashed line) and females (red symbols/lines). Same data as in Figure 4, but divided by sex. In men, data are presented as mean ± SEM. In women, data are reported as mean ± range.

**
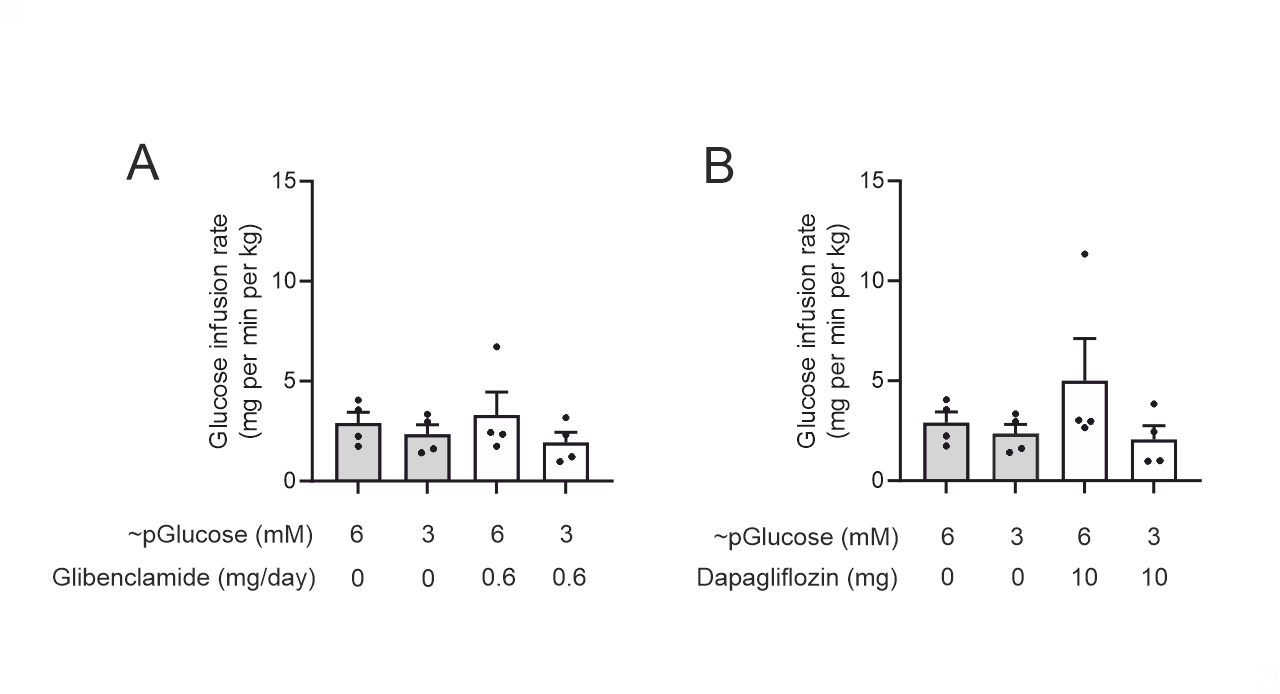
**

**Supplementary Figure 7:** Lack of effects of glibenclamide and dapagliflozin on systemic glucose production in participants with T1D with C-peptide. (A) No effects of glibenclamide (0.6 mg/day) on glucose infusion requirements in Participants with T1D with C-peptide. (B) Same as (A) but after treatment with dapagliflozin (10 mg). Abbreviation: ~pGlucose, approximate plasma glucose concentration (see Figure 3).

**
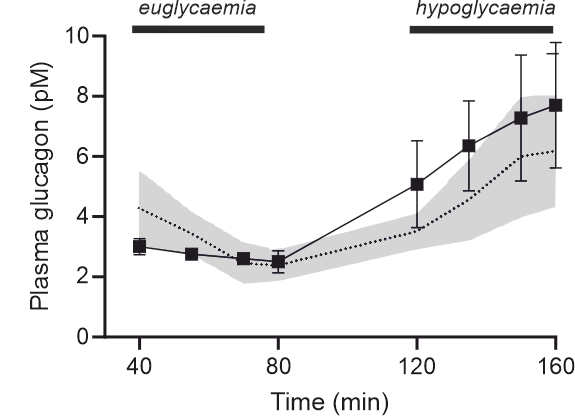
**

**Supplemental Figure 8: No effects of dapagliflozin on plasma glucagon in Participants with T1D with C-peptide.** (A-C) Plasma glucagon levels under hypoglycaemic conditions in participants with T1D and C peptide ≥30 pM during eu- and hypoglycaemic states when treated with dapagliflozin (10 mg, given acutely 2 hours before the clamp). The dotted line and grey area represent mean values ± S.E.M. under basal conditions (same data as in Figure 4C).
