# Supplementary material for: Partial restoration of counterregulatory circulating glucagon by dapagliflozin and low-dose glibenclamide in men with type 1 diabetes: in vitro studies and randomised clinical crossover trial
Source: eBioMedicine. 2026 May 19;128:106298. doi: 10.1016/j.ebiom.2026.106298 (PMC13213787; doi:10.1016/j.ebiom.2026.106298)
Supplement: Statistical Analysis [file mmc2.pdf]

DTU Statistics Group
Oxford Centre for Diabetes, Endocrinology and Metabolism (OCDEM)
Churchill Hospital
Old Road
Oxford/Headington
OX3 7LJ

Statistical Analysis Plan for LEGEND-D

Protocol Title: Low dose glibenclamide and dapagliflozin in Type 1 Diabetes Mellitus

IRAS Project ID: 1004710

Version: 1.0

Date: 2024-11-21

Prepared By:	Prepared For:
[Hudson Mumbole (DTU/SMG)]	*[Sponsor]*

DTU Statistics Group
Oxford Centre for Diabetes, Endocrinology and Metabolism (OCDEM)
Churchill Hospital
Old Road
Oxford/Headington
OX3 7LJ

DIABETES TRIALS UNIT
The Oxford Centre for Diabetes, Endocrinology and Metabolism
UNIVERSITY OF OXFORD

SAP APPROVAL

Signature of Approval for Statistical Analysis Plan for LEGEND-D

**DTU Statistician**	Hudson Mumbole	
	H Mumbole<br>21/Nov/2024 14:51:46<br>Hudson Mumbole	21/11/2024
	Signature	Date
**Trial Statistician**	Mariagrazia Zottoli	
	M Zottoli<br>21/Nov/2024 14:50:29<br>Mariagrazia Zottoli	21/11/2024
	Signature	Date
**Head of SMG (DTU)**	Ruth Coleman	
	R Coleman<br>21/Nov/2024 15:17:44<br>[illegible]	21/11/2024
	Signature	Date
**Clinical Lead**	Ioannis Spilliotis	
	I Spiliotis<br>21/Nov/2024 16:27:34<br>Ioannis Spiliotis	21/11/2024
	Signature	Date

DTU Statistics Group
Oxford Centre for Diabetes, Endocrinology and Metabolism (OCDEM)
Churchill Hospital
Old Road
Oxford/Headington
OX3 7LJ

TABLE OF CONTENT

1 Introduction ... 7
1.1 Study Objectives ... 8
1.1.1 Primary Objectives ... 8
1.1.2 Exploratory Objectives ... 8
1.1.3 Safety Objectives ... 9
1.2 Study Design ... 9
1.3 Participants Identification ... 11
1.3.1 Trial Participants ... 11
1.3.2 Inclusion Criteria ... 11
1.3.3 Exclusion Criteria ... 12
1.3.4 Sample Size Determination ... 13
1.4 Schedule of Study Assessments ... 14
1.5 Changes to the Planned Analysis ... 14

2 Endpoints ... 14
2.1 Primary Endpoints ... 14
2.1.1 Definition and Derivation of Primary Endpoint(s) ... 14
2.1.2 Primary Hypothesis under Investigation ... 14
2.1.3 Stopping Rules ... 15
2.1.4 Handling of Missing Data ... 15
2.2 Exploratory Endpoints ... 15
2.2.1 Definitions and Derivation of Exploratory Endpoints ... 15
2.2.2 Definitions and Derivation of Safety Endpoint(s) ... 15
2.2.3 Handling of Missing Data ... 15

3 Analysis Sets/Populations ... 15
3.1 Protocol violations ... 15
3.2 Intent-to-treat Population / Full analysis set ... 16
3.3 Per-protocol Population ... 16
3.4 Safety Population ... 16
3.5 Other Analysis Populations ... 16

4 General Issues for Statistical Analysis ... 16
4.1 Analysis Software ... 16
4.2 Multiplicity, Multiple Comparisons and Interim Analyses ... 16
4.3 Planned Subgroup Analyses ... 16
4.4 Derived variables ... 16

5 Statistical Methodology ... 16
5.1 Disposition of Patients ... 17
5.2 Baseline Characteristics ... 17
5.3 Primary Endpoint Analysis ... 17
5.3.1 Primary Efficacy Analysis ... 17
5.3.2 Sensitivity ... 18
5.3.3 Subgroup Analyses ... 18
5.4 Exploratory endpoint analyses ... 18
5.4.1 Analysis of exploratory endpoints ... 18
5.4.2 Sensitivity and Exploratory Analyses ... 20
5.4.3 Subgroup Analyses ... 20

5.4.4 Safety Analysis ... 20
5.5 Laboratory Data ... 21

6 Data Handling Conventions ... 21
6.1 Data Monitoring ... 21
6.2 Data Transformations ... 21

DTU Statistics Group
Oxford Centre for Diabetes, Endocrinology and Metabolism (OCDEM)
Churchill Hospital
Old Road
Oxford/Headington
OX3 7LJ

7 References 22

8 Appendices 23
8.1 Appendix 1: Reporting Conventions 23
8.2 Appendix 3: Templates for Tables, Listings and Figures 24
9 Document history 25

DTU Statistics Group
Oxford Centre for Diabetes, Endocrinology and Metabolism (OCDEM)
Churchill Hospital
Old Road
Oxford/Headington
OX3 7LJ

LIST OF ABBREVIATIONS

AE	Adverse Event
AR	Adverse Reaction
AUC	Area Under Curve
α-cells	Alpha-cells
βhCG	beta human Chorionic Gonadotrophin
CGM	Continuous Glucose Monitor
CI	Chief Investigator
CRA	Clinical Research Associate (Monitor)
CRF	Case Report Form
CRO	Contract Research Organisation
CRU	Clinical Research Unit
CT	Clinical Trials
CTA	Clinical Trials Authorisation
DBP	Diastolic Blood Pressure
DEPTH	Dapagliflozin during Exercise for the PrevenTion of Hypos
DM	Diabetes Mellitus
DMC/DMSC	Data Monitoring Committee / Data Monitoring and Safety Committee
DSUR	Development Safety Update Report
DTU	Diabetes Trials Unit
δ-cells	Delta-cells
EPR	Electronic Patient Record
eTMF	Electronic Trial Master File
FGM	Flash Glucose Monitoring
GCP	Good Clinical Practice
GP	General Practitioner
HRA	Health Research Authority
HTA	Human Tissue Authority
ICF	Informed Consent Form
ICH	International Council for Harmonisation
IMP	Investigational Medicinal Product
IRB	Independent Review Board
KATP	ATP-sensitive Potassium channel

DTU Confidential

DTU Statistics Group
Oxford Centre for Diabetes, Endocrinology and Metabolism (OCDEM)
Churchill Hospital
Old Road
Oxford/Headington
OX3 7LJ

LEGEND-A	Low-dose Glibenclamide in Diabetes – Part A
LEGEND-D	Low-dose Glibenclamide and Dapagliflozin in type 1 Diabetes
MHRA	Medicines and Healthcare products Regulatory Agency
mmol/mol	millimoles per mole
ng/ml	nanograms per millilitre
NHS	National Health Service
Non-DM	Without diabetes
RES	Research Ethics Service
RGEA	Research Governance, Ethics and Assurance
OCDEM	Oxford Centre for Diabetes, Endocrinology and Metabolism
OUH	Oxford University Hospitals
pmol	Picomoles
PI	Principal Investigator
PIL	Participant/ Patient Information Leaflet
POC	Point of Care
R&D	NHS Trust R&D Department
REC	Research Ethics Committee
RSI	Reference Safety Information
SAE	Serious Adverse Event
SAR	Serious Adverse Reaction
SBP	Systolic Blood Pressure
SDV	Source Data Verification
SGLT2i	Sodium-glucose co-transporter 2 inhibitor
SMG	Statistics and Modelling Group
SmPC	Summary of Medicinal Product Characteristics
SOP	Standard Operating Procedure
SUSAR	Suspected Unexpected Serious Adverse Reactions
$t_{1/2}$	half-life
TEAE	Treatment Emergent Adverse Events
T1D	Type 1 Diabetes
TMF	Trial Master File
TSG	Oxford University Hospitals NHS Foundation Trust / University of Oxford Trials Safety Group
USAN	United States Adopted Name

DTU Statistics Group
Oxford Centre for Diabetes, Endocrinology and Metabolism (OCDEM)
Churchill Hospital
Old Road
Oxford/Headington
OX3 7LJ

1 Introduction

Type 1 diabetes (T1D) affects around 400,000 people in the UK and is caused by nearly complete loss of insulin-producing cells in the pancreas. It is often challenging to achieve good control of blood glucose throughout the day in T1D, in part because of fluctuations associated with giving insulin. Severe episodes of low blood sugar levels, commonly called “hypos”, are one of the most feared complications of managing diabetes with insulin.

The impaired release of other hormones, such as plasma glucagon which raises blood sugar, during hypo episodes is also part of type 1 diabetes. A better understanding of this mechanism could lead to treatments aimed at reducing the risk of hypoglycaemia.

Glibenclamide is an oral anti-diabetic medication (sulfonylurea) which is commonly used to increase the amount of insulin released by the pancreatic beta-cells. Recent pre-clinical studies have shown that sulfonylureas can also improve plasma glucagon levels when used in very small doses by working on pancreatic alpha-cells, which release plasma glucagon. We have previously conducted a pilot study (LEGEND-A), which suggested that low doses of glibenclamide (0.3mg/day) could alter plasma glucagon release in some people with type 2 diabetes without increasing the risk of hypoglycaemia. In addition, another type of oral anti-diabetic medication, called dapagliflozin, has also been shown to work on pancreatic alpha-cells.

Therefore, the aim of this follow-up study (LEGEND-D) is to find out whether similar doses of glibenclamide or a single dose of dapagliflozin could increase plasma glucagon release in people with T1D. We hope that add-on therapies such as these may become a new way of helping people with T1D to prevent hypoglycaemia.

The trial will involve 2 groups of participants:

a) 20 people with T1D, who will be given a liquid form of glibenclamide orally for a maximum of 54 days (at 3 different doses, in 3 blocks of 14-18 days), followed by a single oral dose of dapagliflozin, and undergo 5 controlled hypoglycaemia challenges.

b) A control group of 10 people without diabetes, who will undergo one hypoglycaemia challenge without receiving any medication.

During these challenges, we will gradually drop participants’ blood sugar from a normal level (around 6 mmol/L) to a lower level (around 2.5mmol/L) for 40 minutes. While this is a well-established procedure, this change in blood sugar can be stressful and participants without diabetes will probably not have experienced the symptoms before. We will screen all participants for high risk conditions, and monitor them closely during

the entire procedure. We will use a continuous glucose monitor during the study in participants with T1D. All participants will need to attend the OCDEM Clinical Research Unit at the Churchill Hospital, Oxford for an initial screening visit. This will be followed by 8 study visits over a period of 8-10 weeks (divided into around 2-week blocks) for the people with T1D, and the people without diabetes will have just 1 study visit.

The purpose of this statistical analysis plan (SAP) is to outline the planned analyses to be performed in the LEGEND-D trial. The SAP will be amended if changes to the planned analyses are required, and will be finalised before the database lock for this study. Exploratory post-hoc or unplanned analyses not necessarily identified in this SAP may be performed on these data as required. These analyses will be clearly identified in the statistical report for the study.

1.1 Study Objectives

1.1.1 Primary Objectives

1. To determine whether treating people with T1D for 14 to 18 days with oral glibenclamide at 0.3mg, 0.6mg or 3mg daily can increase the counter-regulatory plasma glucagon response during induced hypoglycaemia compared to baseline (pre-treatment).

1.1.2 Exploratory Objectives

1. To compare the plasma glucagon response during induced hypoglycaemia in people with T1D prior to glibenclamide treatment, and at each dose step, to that of participants without diabetes (non-DM group).
2. To compare the increase in plasma glucagon from euglycaemia to hypoglycaemia in T1D participants prior to treatment, and at each dose step of glibenclamide, to that of participants without diabetes (non-DM group).
3. To compare the percentage of time spent in hypoglycaemia (

Table *1*) at each dose step of glibenclamdie, compared to baseline (T1D group only)

Table 1 : Definition of hypoglycaemia

**Level 1**	< 4.0 mmol/L (70mg/dL) = **glucose alert**
**Level 2**	< 3.0 mmol/L (54mg/dL) = **clinically important hypoglycaemia**
**Level 3**	<4.0 mmol/L with severe cognitive impairment requiring external assistance for recovery = **severe hypoglycaemia**

4.To compare the change in plasma glucagon from euglycaemia to hypoglycaemia in T1D participants prior to and following administration of a single 10 mg dose of oral dapagliflozin, to that of participants without diabetes (non-DM group).
5.To compare the change in plasma somatostatin from euglycaemia to hypoglycaemia in T1D participants prior to and following administration of a single dose of dapagliflozin 10mg, to that of participants without diabetes (non-DM group).
6.To characterise the development of hypoglycaemic symptoms during induced hypoglycaemia at baseline, and during each dose of glibenclamide and dapagliflozin.
7.To measure any residual C-peptide participants with T1D only.

1.1.3 Safety Objectives

1. To measure the frequency of adverse events in participants treated with Glibenclamide and Dapagliflozin.

1.2 Study Design

The LEGEND-D trial is a pilot, randomised cross-over, single-centre, non-blinded, clinical trial which aims to investigate the effect of 3 low doses of glibenclamide (0.3mg, 0.6mg and 3mg/day) assigned in all possible sequences followed by dapagliflozin (single dose of 10mg) on the plasma glucagon response during induced hypoglycaemia in people with type 1 diabetes, compared to non-diabetic individuals (**Figure 1**). The trial will be conducted by the Oxford Centre for Diabetes, Endocrinology and Metabolism (OCDEM) Clinical Research Unit (CRU) at the Churchill Hospital.

DTU Statistics Group
Oxford Centre for Diabetes, Endocrinology and Metabolism (OCDEM)
Churchill Hospital
Old Road
Oxford/Headington
OX3 7LJ

Figure 1: Trial flow chart

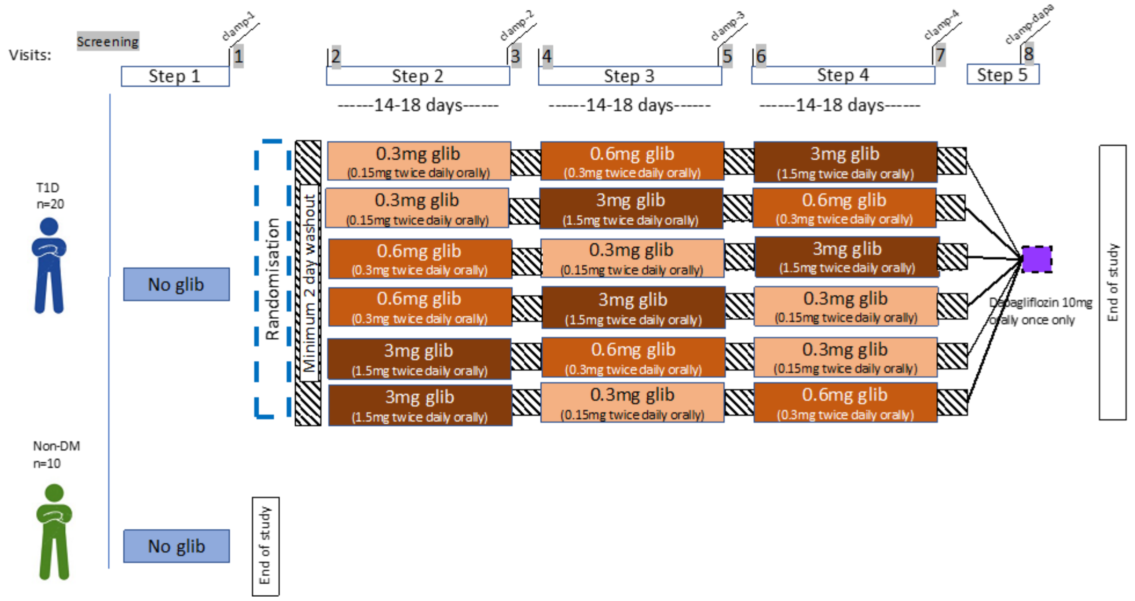

A summary timeline of the trial is shown on figure 1 above. For participants with T1D, the trial is divided into 5 steps, and involves 5 hyperinsulinaemic hypoglycaemic clamps. Once step 1 (no medication) has been completed, the participants with T1D will be randomised to a sequence of glibenclamide doses for the subsequent steps, followed by a single dose of 10mg dapagliflozin. There will be a washout phase of at least 48 hours between each step (the half-life of the glibenclamide suspension is 8 hours) [1]. Participants without diabetes will undergo a single hyperinsulinaemic hypoglycaemic clamp, as they will not receive any study medication.

The total daily dose as defined by the dosing schedule will be split into AM and PM as shown in
Table *2*.

Table 2 : Oral glibenclamide suspension doses and volumes

Total dose	Strength	AM	PM	Total volume over 14 days (min)	Total volume over 18 days (max)
0.3mg	0.6mg/ml	0.25ml	0.25ml	7ml	9ml
0.6mg	0.6mg/ml	0.5ml	0.5ml	14ml	18ml
3mg	0.6mg/ml	2.5ml	2.5ml	70ml	90ml
			**Total:**	**91ml**	**117ml**

DTU Statistics Group
Oxford Centre for Diabetes, Endocrinology and Metabolism (OCDEM)
Churchill Hospital
Old Road
Oxford/Headington
OX3 7LJ

As mentioned above, for participants with T1D the final hyperinsulinaemic hypoglycaemic clamp will involve them taking a single dose of dapagliflozin 10mg orally at the start of the study visit.

The main pharmacologically relevant site of action of dapagliflozin (Forxiga, AstraZeneca), is the sodium-glucose cotransporter 2 in the proximal convoluted tubules of the kidneys. By inhibiting this transporter, the threshold for glucose reabsorption is reduced, leading to increased glucose excretion.

Dapagliflozin also leads to increased plasma glucagon secretion by acting directly on the pancreatic islet cells [2]. Plasma glucagon concentrations are increased compared to baseline 2 hour after dapagliflozin administration, and reach a peak of 79pg/ml (23.7pmol/L) after 4 hours [3].

In total there will be 8 study visits for participants with T1D and 1 study visit for the non-DM participants.

1.3 Participants Identification

1.3.1 Trial Participants

The anticipated recruitment period is 12 months; the recruitment target is 20 participants with T1D and 10 non-DM controls. The study will aim to include participants of different age groups.

1.3.2 Inclusion Criteria

Type 1 diabetes group:

1. T1D diagnosed ≥12 months prior to screening
2. Age 18-75 years
3. Either on insulin pump or multiple daily insulin injections
4. HbA1c < 10% (86mmol/mol) at screening
5. Prior training regarding insulin dose-adjustment and management of hypoglycaemia
6. Willing and able to give informed consent for participation in the trial

DTU Statistics Group
Oxford Centre for Diabetes, Endocrinology and Metabolism (OCDEM)
Churchill Hospital
Old Road
Oxford/Headington
OX3 7LJ

7. In the Investigator's opinion is able and willing to comply with all trial requirements

Non-DM control group**:**

1. Age 18-75 years
2. HbA1c ≤6.0% (42mmol/mol) at screening
3. Willing and able to give informed consent for participation in the trial
4. In the Investigator's opinion is able and willing to comply with all trial requirements

1.3.3 Exclusion Criteria

T1D group only:

1. An episode of diabetic ketoacidosis in the previous 1 month
2. Severe hypoglycaemia requiring third party intervention on more than 1 occasion in the preceding 12 months
3. Active diabetic retinopathy (including active proliferative diabetic retinopathy or vitreous haemorrhage in the past 6 months)

T1D and non-diabetic group:

4. Haemoglobin < 125 g/L
5. History of seizure or coma
6. Pregnancy, breast feeding or women of childbearing potential without adequate contraception
7. Renal impairment (eGFR ≤50 ml/min) at screening
8. ALT >2.5×upper limit of the assay normal range or known liver disease, specifically bilirubin >30 μmol/L that is associated with other evidence of liver failure.
9. Uncontrolled hypertension (>180 mmHg systolic or > 100 mmHg diastolic)
10. History of ischaemic heart disease (unless has had successful reperfusion), stroke/transient ischaemic attack (TIA), ventricular rhythm disturbances or thromboembolic disease
11. On beta-blocker medication
12. A history of heart failure (New York Heart Association, NYHA, Class 3 or 4)

DTU Statistics Group
Oxford Centre for Diabetes, Endocrinology and Metabolism (OCDEM)
Churchill Hospital
Old Road
Oxford/Headington
OX3 7LJ

13. Untreated Grave's disease

14. History of ECG or stress test findings indicating active ischaemia or a condition that would compromise the participant's safety

15. Known history of porphyria

16. Concomitant use of bosentan

17. Known or suspected allergy to the trial product or related products

18. Have received any investigational drug within 3 months prior to screening

19. Systemic (i.e. other than topical) corticosteroid treatment within 30 days prior to the start or at any time during the trial period

20. Major psychiatric disease including eating disorders, history of drug and alcohol abuse

21. Known malignancy or any other condition or circumstance which, in the opinion of the investigator, would affect the participant's ability to participate in the protocol

It is worth noting that although the SmPC of both Amiglida (glibenclamide) and Forxiga (Dapagliflozin) present an extensive list of medications which might interact with these drugs, given the low dosage administration, the limited number of times that participants are required to administer these medications, as well as the different study population investigated, these interactions are not considered clinically relevant and will not form part of the exclusion criteria.

1.3.4 Sample Size Determination

There are currently no data on plasma glucagon levels from human trials using low-dose sulfonylureas in patients with type 1 diabetes. The sample size calculations are based on the data generated from the LEGEND-A trial [4] (NCT02830048 - completed) and interim analysis from the DEPTH trial (NCT03537131 – suspended due to the COVID-19 pandemic).

The LEGEND-A trial suggested glibenclamide (at a dose of 0.3mg/day) could change plasma glucagon levels in participants with type 2 diabetes by 30% (magnitude of effect), while the DEPTH trial suggested that participants with type 1 diabetes who experienced exercise-induced hypoglycaemia had mean plasma glucagon values of 5.54pmol/L (SD 2.14pmol/L) at the time of hypoglycaemia (defined as <3.3mmol/L).

DTU Statistics Group
Oxford Centre for Diabetes, Endocrinology and Metabolism (OCDEM)
Churchill Hospital
Old Road
Oxford/Headington
OX3 7LJ

For the non-diabetic control group, data from a clinical study in healthy volunteers [5], which used similar methodology and a plasma glucagon assay comparable to the one we will use in our trial, demonstrated that the mean plasma glucagon values during the hypoglycaemia plateau of 23.3 pmol/L (SD 5.7pmol/L).

These figures indicate that 20 participants with T1D, and 10 participants without diabetes (including 5% dropout) would give the LEGEND-D trial 80% power to detect a 30% increase in plasma glucagon levels (the primary endpoint) during induced hypoglycaemia (hyperinsulinaemic-hypoglycaemic clamp).

1.4 Schedule of Study Assessments

Individual participant’s involvement: T1D group = maximum 77 days, including 8 days washout non-DM group = 1 day. Trial duration: 11 months

Trial start date: 15 August 2023

Trial End Date: 30 May 2025

Estimated recruitment dates: August’23 – 30 November 2024

1.5 Changes to the Planned Analysis

No intended changes to planned analysis.

2 Endpoints

2.1 Primary Endpoints

2.1.1 Definition and Derivation of Primary Endpoint(s)

The primary endpoint in this study is the difference in plasma glucagon response during induced hypoglycaemia and baseline (pre-treatment). It is defined as the concentration of plasma glucagon measured in pmol/L at 40min during the hypoglycaemic phase of the hyper-insulinaemic hypoglycaemic clamp, at baseline and at each dose step i.e. 0.3mg, 0.6mg or 3mg of glibenclamide (T1D group only).

2.1.2 Primary Hypothesis under Investigation

The hypothesis to be tested for the primary analysis is given below:
H_0: There is no difference in counter-regulatory plasma glucagon concentration (pmol/L) during induced hypoglycaemia compared to baseline (pre-treatment) in people randomised to glibenclamide at 0.3mg, 0.6mg or 3mg (**Table 3**) daily for 14-18 days.

DTU Statistics Group
Oxford Centre for Diabetes, Endocrinology and Metabolism (OCDEM)
Churchill Hospital
Old Road
Oxford/Headington
OX3 7LJ

2.1.3 Stopping Rules

The trial will be terminated if there are serious adverse events that occur in three or more (>10%) of participants.

2.1.4 Handling of Missing Data

Any missing data regarding blood results will be checked with the corresponding labs (Oxford University Hospitals NHS Foundation Trust or University of Oxford). The same will apply to spurious data. If two or more data points for the primary outcome (glucagon concentration) are missing from the same participant (excluding the baseline hypo clamp, which is essential), then the participant will be withdrawn from the trial.

2.2 Exploratory Endpoints

2.2.1 Definitions and Derivation of Exploratory Endpoints

Exploratory efficacy endpoints are defined as the difference between measurements (defined below) at baseline and at post treatment period (i.e. 0, 15, 30 and 40min):
- Plasma glucagon level is the concentration of plasma glucagon measured in pmol/L.
- Plasma somatostatin level is the concentration of plasma somatostatin measured in pmol/L.
- Blood glucose value is the concentration of blood glucose measured in mmol/L.
- Plasma C-peptide level is the concentration of plasma C-peptide measured in pmol/L.

2.2.2 Definitions and Derivation of Safety Endpoint(s)

Safety endpoint is defined as number of adverse events during treatment period of glibenclamide (at 0.3mg, 0.6mg or 3mg) and dapagliflozin **Table 4**.

2.2.3 Handling of Missing Data

All missing data will be handled as detailed in 2.1.4.

3 Analysis Sets/Populations

3.1 Protocol violations

A trial-related deviation is a departure from the ethically approved trial protocol or other trial document or process (e.g. consent process or IMP administration) or from Good Clinical Practice (GCP) or any applicable regulatory requirements. Any deviations from the protocol will be documented in a protocol deviation form and filed in the trial master file.

DTU Statistics Group
Oxford Centre for Diabetes, Endocrinology and Metabolism (OCDEM)
Churchill Hospital
Old Road
Oxford/Headington
OX3 7LJ

3.2 Intent-to-treat Population / Full analysis set

The intention to treat population (ITT) is defined as the set of all randomised patients. Any patient found to have followed a sequence that is different from that to which they were randomised will be counted in the sequence to which they were randomised. Hence, data from all randomised participants will be included in the analysis.

3.3 Per-protocol Population

The per-protocol population (PP) is defined as the set of all randomised patients that adhered to the protocol. There are no PP analyses.

3.4 Safety Population

Safety population (SP) analysis is defined as the set of all randomised patients who were treated with glibenclamide and dapagliflozin.

3.5 Other Analysis Populations

No other analysis populations are planned at this stage

4 General Issues for Statistical Analysis

4.1 Analysis Software

All analyses will be performed using a statistical package SAS/R/STATA.

4.2 Multiplicity, Multiple Comparisons and Interim Analyses

There will be no multiplicity adjustment for this trial.

4.3 Planned Subgroup Analyses

No subgroup analyses have been planned.

4.4 Derived variables

Body mass index (BMI) variable will be derived from height and weight of participants using the formula below:

• BMI = Weight (kg) / (Height (m)) ^{2}.

5 Statistical Methodology

The statistical methodology for the main primary analysis is described below. Analyses will use the ITT population (section 3.2) for primary and exploratory endpoint(s), and safety population for safety endpoint(s). All statistical significance will be assessed using

DTU Statistics Group
Oxford Centre for Diabetes, Endocrinology and Metabolism (OCDEM)
Churchill Hospital
Old Road
Oxford/Headington
OX3 7LJ

an alpha value of 0.05 (95% confidence interval). The summary statistics and statistical estimates will be presented with one decimal place unless more decimal places are needed to interpret the data. All p-values will be rounded to 3 decimal places. All Tables and Figures documented in this SAP are shell versions of the anticipated results and output formats of the analyses to be conducted for the study.

5.1 Disposition of Patients

Enrolment according to the population described in section 3.2 will be summarised and listed. The rate and relevant reasons for discontinuation will be described by treatment and period.

5.2 Baseline Characteristics

The demographic, clinical and anthropometric characteristics as well as the medical history data will be tabulated and summarised. We will use descriptive statistics [counts (percentage) for categorical variables and mean (standard deviation) or median (interquartile range) for continuous variables] to summarize baseline characteristics of participants, as well as outcome variables for each treatment period. When a variable is measured over time it will be summarised by period. The demographic data will include age, gender, body weight, height, SBP, DBP, resting heart rate, HbA1c, FBC, U&E, LFTs, Blood Glucose, Plasma glucagon, Plasma C-peptide and Plasma somatostatin by diabetes status (T1D/non-DM) **Table 5**.

5.3 Primary Endpoint Analysis

5.3.1 Primary Efficacy Analysis

To determine whether people randomised to glibenclamide at 0.3mg, 0.6mg or 3mg daily for 14-18 days can change the counter-regulatory plasma glucagon concentration measured in pmol/L at 40min during induced hypoglycaemia compared to baseline (pre-treatment) in T1D group only. We will use 3 separate paired t-tests to compare plasma glucagon concentration (pmol/L) at baseline and at each glibenclamide dose step i.e. 0mg vs 0.3mg, 0mg vs 0.6mg, and 0mg vs 3mg. We will also use linear random effects model [6] using plasma glucagon concentration (pmol/L) at each glibenclamide dose step as dependent variable and including treatment, period, and sequence of treatment to account for within-subject correlation to quantify the magnitude and direction of the change (increase/decrease). We will also adjust for age, gender, body weight, height, SBP, DBP and resting heart rate.

5.3.2 Sensitivity

No sensitivity analysis is planned for primary endpoint.

5.3.3 Subgroup Analyses

No subgroup analyses are planned.

5.4 Exploratory endpoint analyses

5.4.1 Analysis of exploratory endpoints

1. To compare the concentration of plasma glucagon measured in pmol/L during induced hypoglycaemia (at 0, 15, 30 and 40min) in people with T1D prior to glibenclamide treatment, and at each dose step (at 0.3mg, 0.6mg and 3mg), to that of participants without diabetes (non-DM group).

A mixed-effects model (treatment x time-point) will be used to compare plasma glucagon values at each time-point (0, 15, 30 and 40min) during the hypoglycaemic phase of the hyperinsulinaemic hypoglycaemic clamp at baseline (T1D and non-DM group) and at each dose step (T1D group only).

Post-hoc analysis will be performed which will allow pairwise comparisons between the plasma glucagon values obtained at each time-point (T1D and non-DM group, see **Table 3**).

2. To compare the proportion of change in plasma glucagon concentration from euglycaemia to hypoglycaemia at each time point (0, 15, 30 and 40min) in T1D participants prior to treatment, and at each dose step (at 0.3mg, 0.6mg and 3mg) of glibenclamide, to that of participants without diabetes (non-DM group).
We will calculate the proportion of increase in plasma glucagon at each time point by taking plasma glucagon values at each time-point (0, 15, 30 and 40min) during the hypoglycaemic phase of the hyperinsulinaemic hypoglycaemic clamp divided by the value at the corresponding time-point during the euglycaemic phase.

A mixed-effects model (treatment x time-point) will be used to compare the proportion increase in plasma glucagon as dependent variable (i.e. plasma glucagon concentration during the hypoglycaemic phase vs plasma glucagon concentration during the euglycaemic phase) at each time-point (0, 15, 30 and 40min) as independent variable at baseline (T1D and non-DM group) and at each dose step (T1D group only).

DTU Statistics Group
Oxford Centre for Diabetes, Endocrinology and Metabolism (OCDEM)
Churchill Hospital
Old Road
Oxford/Headington
OX3 7LJ

Post-hoc analysis will be performed which will allow pairwise comparisons between the plasma glucagon values obtained at each time-point (see **Table 3**).

3. To compare the percentage of time spent in hypoglycaemia

Table *1* (<4.0mmol/L of blood glucose) at each dose step (at 0.3mg, 0.6mg and 3mg) of glibenclamide, compared to baseline (T1D group only). Three separate paired t-tests will be used to compare 0mg vs 0.3mg, 0mg vs 0.6mg and 0mg vs 3mg. If the assumptions of t-test are not satisfied, then the percentages will be treated as proportion and a 2 proportions test will be used.

4. To compare the proportion of change in plasma glucagon levels measured in pmol/L at each time point 0, 15, 30 and 40min, (i.e. glucagon concentration at 0min during the hypoglycaemic phase divided by the glucagon concentration at 0min during the euglycaemic phase) from euglycaemia to hypoglycaemia in T1D participants prior to and following administration of a single 10 mg dose of oral dapagliflozin, to that of participants without diabetes (non-DM group).

Plasma glucagon values at each time-point (0, 15, 30 and 40min) during the hypoglycaemic phase of the hyperinsulinaemic hypoglycaemic clamp will be divided by the value at the corresponding time-point during the euglycaemic phase. A mixed-effects model (treatment x time-point) will be used to compare the proportion change in plasma glucagon (i.e.plasma glucagon during the hypoglycaemic phase vs plasma glucagon during the euglycaemic phase) after taking a single dose of dapagliflozin (T1D group only) versus baseline/no treatment (T1D and non-DM group).

Post-hoc analysis will be performed which will allow pairwise comparisons between the plasma glucagon values obtained at each time-point.

5. To compare the proportion of change in plasma somatostatin levels measured in pmol/L at each time point 0, 15, 30 and 40min (i.e. somatostatin concentration at 0min during the hypoglycaemic phase divided by the somatostatin concentration at 0min during the euglycaemic phase) from euglycaemia to hypoglycaemia in T1D participants prior to and following administration of a single dose of dapagliflozin 10mg, to that of participants without diabetes (non-DM group).

DTU Statistics Group
Oxford Centre for Diabetes, Endocrinology and Metabolism (OCDEM)
Churchill Hospital
Old Road
Oxford/Headington
OX3 7LJ

Plasma somatostatin values at each time-point (0, 15, 30 and 40min) during the hypoglycaemic phase of the hyperinsulinaemic hypoglycaemic clamp will be divided by the value of plasma somatostatin at the corresponding time-point during the euglycaemic phase.

A mixed-effects model (treatment x time-point) will be used to compare the proportion change in somatostatin (i.e. somatostatin during the hypoglycaemic phase vs. somatostatin during the euglycaemic phase) after taking a single dose of dapagliflozin (T1D group only) versus baseline/no treatment (T1D and non-DM group).

Post-hoc analysis will be performed which will allow pairwise comparisons using correlation [7] between the plasma somatostatin values obtained at each time-point.

6**.** To characterise the development of hypoglycaemic symptoms by listing self-reported hypoglycaemia symptoms and recording blood glucose value (mmol/L) at onset of self-reported hypoglycaemia symptoms (see **Table 4Error! Reference source not found.**: Assessment of hypoglycaemia symptoms) during the induced hypoglycaemia at baseline, and during each dose of glibenclamide (at 0.3mg, 0.6mg or 3mg) and dapagliflozin.

A multilevel modelling approach (treatment x group) will be used to compare the onset of self-reported hypoglycaemic symptoms during the hyperinsulinaemic hypoglycaemic clamp between the group with and without diabetes.

7. To measure fasting plasma C-peptide levels (pmol/L) at the start of the hyperinsulinaemic hypoglycaemic clamp (T1D group only).

We will use descriptive statistics to describe fasting plasma C-peptide levels (pmol/L) at the start of the hyperinsulinaemic hypoglycaemic clamp (T1D group only). This will be in the form of mean (standard deviation) if data is normally distributed or median (interquartile range) if not normally distributed.

5.4.2 Sensitivity and Exploratory Analyses

Exploratory analyses for efficacy will be carried out. No sensitivity analysis is planned for exploratory endpoints.

5.4.3 Subgroup Analyses

No subgroup analyses are planned.

5.4.4 Safety Analysis

1. To measure the frequency of adverse events in participants treated with Glibenclamide and Dapagliflozin at each study visit.

Descriptive statistics in form of percentages will be used to determine the proportion of adverse events.

Summary tables for adverse events will include the following:

- Counts (percentage) of subjects with any AEs by treatment and sequence.
- Counts (percentage) with any TEAEs by treatment and sequence.
- Counts (percentage) of subjects with any related TEAEs (AR) by treatment and sequence
- Counts (percentage) of subjects with any serious TEAEs (SAE) by treatment and sequence
- Counts (percentage) of subjects with any related serious TEAEs (SAR) by treatment and sequence

5.5 Laboratory Data

All laboratory results such as plasma somatostatin concentration, plasma glucagon concentration, Haemoglobin, white cell count, haematocrit and platelets etc. will be reviewed and the reports signed by a qualified member of the clinical research team, who will record in the CRF whether it is normal, abnormal but not clinically significant, or abnormal and clinically significant.

The Freestyle Libre data will be collected, stored and retrieved in accordance with the privacy policy of Freestyle Libre web site controlled by Abbott Laboratories. The cloud-based diabetes management system has been assessed and approved by the University of Oxford Information Security Team.

6 Data Handling Conventions

6.1 Data Monitoring

Regular monitoring will be performed according to the trial specific Monitoring Plan. Data will be evaluated for compliance with the protocol and accuracy in relation to source documents as these are defined in the trial specific Monitoring Plan. Following written standard operating procedures, the monitors will verify that the clinical trial is conducted and data are generated, documented and reported in compliance with the protocol, GCP and the applicable regulatory requirements.

6.2 Data Transformations

No data transformations have been identified in this SAP.

7 References

1. Trube G, Rorsman P, Ohno-Shosaku T. Opposite effects of tolbutamide and diazoxide on the ATP-dependent K+ channel in mouse pancreatic beta-cells. Pflugers Arch. 1986;407(5):493-499.
2. Bonner C, Kerr-Conte J, Gmyr V, Queniat G, Moerman E, Thévenet J, Beaucamps C, Delalleau N, Popescu I, Malaisse WJ. Inhibition of the glucose transporter SGLT2 with dapagliflozin in pancreatic alpha cells triggers glucagon secretion. Nature Medicine. 2015;21(5):512-517.
3. Merovci A, Solis-Herrera C, Daniele G, Eldor R, Fiorentino TV, Tripathy D, Xiong J, Perez Z, Norton L, Abdul-Ghani MA. Dapagliflozin improves muscle insulin sensitivity but enhances endogenous glucose production. The Journal of clinical investigation. 2014;124(2):509-514.
4. Spiliotis, II, Chalk R, Gough S, Rorsman P. Reducing hyperglucagonaemia in type 2 diabetes using low-dose glibenclamide: Results of the LEGEND-A pilot study. Diabetes Obes Metab. 2022;24(8):1671-1675.
5. Nauck MA, Heimesaat MM, Behle K, Holst JJ, Nauck MS, Ritzel R, Hufner M, Schmiegel WH. Effects of glucagon-like peptide 1 on counterregulatory hormone responses, cognitive functions, and insulin secretion during hyperinsulinemic, stepped hypoglycemic clamp experiments in healthy volunteers. J Clin Endocrinol Metab. 2002;87(3):1239-1246.
6. Greenland S. Principles of multilevel modelling. Int J Epidemiol. 2000;29(1):158-67.
7. Alison C. Holloway, Mark A. Sheridan, Glen Van Der Kraak, John F. Leatherland, Correlations of plasma growth hormone with somatostatin, gonadal steroid hormones and thyroid hormones in rainbow trout during sexual recrudescence, Comparative Biochemistry and Physiology Part B: Biochemistry and Molecular Biology,Volume 123, Issue 3,1999,Pages 251-260, ISSN 1096-4959.

DTU Statistics Group
Oxford Centre for Diabetes, Endocrinology and Metabolism (OCDEM)
Churchill Hospital
Old Road
Oxford/Headington
OX3 7LJ

8 Appendices

8.1 Appendix 1: Reporting Conventions

Table 3 : Groups for comparison by oral glibenclamide dose in milligrams (mg)

		non-DM	T1D		
		0	0.3	0.6	3
T1D	0	vs.	vs.	vs.	vs.
	0.3	vs.	-	vs.	vs.
	0.6	vs.	-	-	vs.
	3	vs.	-	-	-

Table 4 : Assessment of hypoglycaemia symptoms

**Autonomic**	**Neuroglycopenic**	**Non-specific**
Sweating	Inability to concentrate	Tingling around the mouth
Trembling	Confusion	Dry mouth
Flushing	Tiredness	Blurred vision
Anxiety	Feeling tearful	Headache
Pounding heart	Difficulty in speaking	Nausea
Hunger	Odd behaviour	
	Incoordination	
	Weakness	
	Drowsiness	

8.2 Appendix 3: Templates for Tables, Listings and Figures

Table 5 : Baseline characteristics

Variable	All	T1D	Non-DM
Age in years:			
Mean (SD)	xx (xx)	xx (xx)	xx (xx)
Median (Q1-Q3)	xx (xx-xx)	xx (xx-xx)	xx (xx-xx)
Gender: N (%)			
Male	xx (xx.xx)	xx (xx.xx)	xx (xx.xx)
Female	xx (xx.xx)	xx (xx.xx)	xx (xx.xx)
BMI (kg/m^2):			
Mean (SD)	xx (xx)	xx (xx)	xx (xx)
Median (Q1-Q3)	xx (xx-xx)	xx (xx-xx)	xx (xx-xx)
SBP(mmHg):			
Mean (SD)	xx (xx)	xx (xx)	xx (xx)
Median (Q1-Q3)	xx (xx-xx)	xx (xx-xx)	xx (xx-xx)
DBP (mmHg):			
Mean (SD)	xx (xx)	xx (xx)	xx (xx)
Median (Q1-Q3)	xx (xx-xx)	xx (xx-xx)	xx (xx-xx)
Resting Heart rate (beats per minute):			
Mean (SD)	xx (xx)	xx (xx)	xx (xx)
Median (Q1-Q3)	xx (xx-xx)	xx (xx-xx)	xx (xx-xx)
Blood Glucose (mmol/L):			
Mean (SD)	xx (xx)	xx (xx)	xx (xx)
Median (Q1-Q3)	xx (xx-xx)	xx (xx-xx)	xx (xx-xx)

DTU Statistics Group
Oxford Centre for Diabetes, Endocrinology and Metabolism (OCDEM)
Churchill Hospital
Old Road
Oxford/Headington
OX3 7LJ

Plasma Glucagon (pmol/L):			
Mean (SD)	xx (xx)	xx (xx)	xx (xx)
Median (Q1-Q3)	xx (xx-xx)	xx (xx-xx)	xx (xx-xx)
Plasma C-peptide (pmol/L):			
Mean (SD)	xx (xx)	xx (xx)	xx (xx)
Median (Q1-Q3)	xx (xx-xx)	xx (xx-xx)	xx (xx-xx)
Plasma Somatostatin (pmol/L):			
Mean (SD)	xx (xx)	xx (xx)	xx (xx)
Median (Q1-Q3)	xx (xx-xx)	xx (xx-xx)	xx (xx-xx)
Blood: HbA1c, FBC*, U&E, LFTs.			
Mean (SD)	xx (xx)	xx (xx)	xx (xx)
Median (Q1-Q3)	xx (xx-xx)	xx (xx-xx)	xx (xx-xx)

**Haemoglobin, white cell count, haematocrit and platelets*

9 Document history

Version Number	Effective Date	Significant Changes
0.1	2023-09-19	Initial version
0.2	2024-06-17	Second draft following review by RLC, AIA
0.3	2024-07-11	Third draft following review by RLC, AIA
0.4	2024-10-17	Fourth draft following review by RLC, AIA, IS
1.0	2024-11-21	Final version

RLC-Ruth L. Coleman, AIA-Amanda I. Adler, IS- Ioannis Spilliotis

This Document has been Signed with a **secure electronic signature** via E-Sign.

Envelope Details

**Title**	20241121_LEGEND-D_SAP_v1.0_clean
**Author**	Ada Tse (@ Diabetes Trials Unit) (ada.tse@dtu.ox.ac.uk)
**Envelope Created on**	Thu, 21 Nov 2024 14:47:42
**Envelope ID**	678c8b18-68d5-4538-b065-dfa6cb20460d

Document Details

**Title**	20241121_LEGEND-D_SAP_v1.0_clean
**Digital Fingerprint**	44dc86d9-3701-43a4-b577-e27fb0467c6e

Document Signers Scan/Click the QR Code to view signature information

**Name**	Hudson Mumbole
**Email**	hudson.mumbole@dtu.ox.ac.uk
**Status**	**SIGNED** at Thu, 21 Nov 2024 14:51:46 GMT(+0000)
**Signature Fingerprint**	97f7b141-84c3-4bf7-9a10-0dc1e4674f40

**Name**	Mariagrazia Zottoli
**Email**	mariagrazia.zottoli@stats.ox.ac.uk
**Status**	**SIGNED** at Thu, 21 Nov 2024 14:50:29 GMT(+0000)
**Signature Fingerprint**	a98c61bd-2551-4800-a2f5-1e471e00385e

**Name**	Ruth Coleman
**Email**	ruth.coleman@dtu.ox.ac.uk
**Status**	**SIGNED** at Thu, 21 Nov 2024 15:17:44 GMT(+0000)
**Signature Fingerprint**	f2928490-c7a7-4bbc-8c58-78397d41b71c

**Name**	Ioannis Spiliotis
**Email**	ioannis.spiliotis@ocdem.ox.ac.uk
**Status**	**SIGNED** at Thu, 21 Nov 2024 16:27:34 GMT(+0000)
**Signature Fingerprint**	a956c2dd-65dc-41cb-9d68-c45e9fd1b904

ico. Information Commissioner's Office
British Assessment Bureau UKAS MANAGEMENT SYSTEMS 8289 ISO 27001 INFORMATION SECURITY MANAGEMENT
legislation.gov.uk
DEPARTMENT OF COMMERCE UNITED STATES OF AMERICA

Document History

**Thu, 21 Nov 2024 16:27:34**	**Ioannis Spiliotis** Signed the Document (IP: 82.31.156.58)
**Thu, 21 Nov 2024 15:17:45**	**Ruth Coleman** Signed the Document (IP: 163.1.202.193)
**Thu, 21 Nov 2024 14:51:47**	**Hudson Mumbole** Signed the Document (IP: 163.1.198.214)
**Thu, 21 Nov 2024 14:50:30**	**Mariagrazia Zottoli** Signed the Document (IP: 192.76.8.77)
